# Supplementary material for: Emergence and spread of SARS-CoV-2 lineage B.1.620 with variant of concern-like mutations and deletions
Source: Nat Commun. 2021 Oct 1;12:5769. doi: 10.1038/s41467-021-26055-8 (PMC8486757; doi:10.1038/s41467-021-26055-8)
Supplement: Supplementary file 3 — Reporting Summary [file 41467_2021_26055_MOESM3_ESM.pdf]

nature portfolio

Corresponding author(s): Gytis Dudas

Last updated by author(s): Sep 8, 2021

Reporting Summary

Nature Portfolio wishes to improve the reproducibility of the work that we publish. This form provides structure for consistency and transparency in reporting. For further information on Nature Portfolio policies, see our Editorial Policies and the Editorial Policy Checklist.

Statistics

For all statistical analyses, confirm that the following items are present in the figure legend, table legend, main text, or Methods section.

n/a	Confirmed	
☐	☒	The exact sample size (*n*) for each experimental group/condition, given as a discrete number and unit of measurement
☒	☐	A statement on whether measurements were taken from distinct samples or whether the same sample was measured repeatedly
☒	☐	The statistical test(s) used AND whether they are one- or two-sided<br>*Only common tests should be described solely by name; describe more complex techniques in the Methods section.*
☒	☐	A description of all covariates tested
☒	☐	A description of any assumptions or corrections, such as tests of normality and adjustment for multiple comparisons
☐	☒	A full description of the statistical parameters including central tendency (e.g. means) or other basic estimates (e.g. regression coefficient) AND variation (e.g. standard deviation) or associated estimates of uncertainty (e.g. confidence intervals)
☒	☐	For null hypothesis testing, the test statistic (e.g. *F*, *t*, *r*) with confidence intervals, effect sizes, degrees of freedom and *P* value noted<br>*Give P values as exact values whenever suitable.*
☐	☒	For Bayesian analysis, information on the choice of priors and Markov chain Monte Carlo settings
☒	☐	For hierarchical and complex designs, identification of the appropriate level for tests and full reporting of outcomes
☒	☐	Estimates of effect sizes (e.g. Cohen's *d*, Pearson's *r*), indicating how they were calculated

Our web collection on statistics for biologists contains articles on many of the points above.

Software and code

Policy information about availability of computer code

Data collection	Data downloaded after deposition on GISAID, an acknowledgment table listing all genomes used is available as supplementary information and reachable at https://github.com/evogytis/B.1.620-in-Europe/blob/main/data/acknowledgment_table/gisaid_hcov-19_acknowledgement_table_2021_07_29_10.pdf.
Data analysis	MAFFT v7, PhyML v3.0, BEAST v1.10.5, baltic v0.1.6, TempEst v1.5.3, Tracer v1.7, TreeAnnotator v1.10.5, HADDOCK v2.4, IQ-TREE v1.6.12, custom scripts used to analyse data and produce figures is available at https://github.com/evogytis/B.1.620-in-Europe/tree/main/scripts.

For manuscripts utilizing custom algorithms or software that are central to the research but not yet described in published literature, software must be made available to editors and reviewers. We strongly encourage code deposition in a community repository (e.g. GitHub). See the Nature Portfolio guidelines for submitting code & software for further information.

Data

Policy information about availability of data

All manuscripts must include a data availability statement. This statement should provide the following information, where applicable:

- Accession codes, unique identifiers, or web links for publicly available datasets
- A description of any restrictions on data availability
- For clinical datasets or third party data, please ensure that the statement adheres to our policy

A list of GISAID accessions for genomes used here, as well as phylogenetic trees used in figures are available at https://github.com/evogytis/B.1.620-in-Europe or under Zenodo DOI https://doi.org/10.5281/zenodo.5494346.
To access sequence data from GISAID one has to register an account with https://www.gisaid.org/ which involves identifying oneself and agreeing to GISAID's Database Access Agreement.

Scripts used to generate figures are available at https://github.com/evogytis/B.1.620-in-Europe or under Zenodo DOI https://doi.org/10.5281/zenodo.5494346. We provide the XML files to perform the Bayesian phylogeographic reconstructions in BEAST 1.10.5 as Supplementary Material.

Field-specific reporting

Please select the one below that is the best fit for your research. If you are not sure, read the appropriate sections before making your selection.

- [x] Life sciences
- [ ] Behavioural & social sciences
- [ ] Ecological, evolutionary & environmental sciences

For a reference copy of the document with all sections, see nature.com/documents/nr-reporting-summary-flat.pdf

Life sciences study design

All studies must disclose on these points even when the disclosure is negative.

Sample size	As a phylogenetic study all available high quality B.1.620 genomes were used with as many background sequences as possible to run in reasonable time.
Data exclusions	Some B.1.620 sequences were either excluded on evidence of poor quality like presence of characteristic mutations but not deletions or trimmed when clusters of unique mutations/non-homologous regions were identified at the ends of the genome. Similarly we could not include sequences submitted to GISAID after we submitted the manuscript. As of 2021 September 7 the following GISAID accessions were not included in our analyses for one or more of the reasons stated earlier: EPI_ISL_3229501, EPI_ISL_3147605, EPI_ISL_2887413, EPI_ISL_3545056, EPI_ISL_2710431, EPI_ISL_2710508, EPI_ISL_2710530, EPI_ISL_2978193, EPI_ISL_2710426, EPI_ISL_2967252, EPI_ISL_2710483, EPI_ISL_3369249, EPI_ISL_2710487, EPI_ISL_2968574, EPI_ISL_2710389, EPI_ISL_3451963, EPI_ISL_2710428, EPI_ISL_2940878, EPI_ISL_3544722, EPI_ISL_3674634, EPI_ISL_2710523, EPI_ISL_2710466, EPI_ISL_3544970, EPI_ISL_2710489, EPI_ISL_2710505, EPI_ISL_3138804, EPI_ISL_3545204, EPI_ISL_2710486, EPI_ISL_2694574, EPI_ISL_3061185, EPI_ISL_2281277, EPI_ISL_2710441, EPI_ISL_2710538, EPI_ISL_2790922, EPI_ISL_2967253, EPI_ISL_2710500, EPI_ISL_3452058, EPI_ISL_2889849, EPI_ISL_3040135, EPI_ISL_2710516, EPI_ISL_3026527, EPI_ISL_3040132, EPI_ISL_2889853, EPI_ISL_3237538, EPI_ISL_2710534, EPI_ISL_3475212, EPI_ISL_3451998, EPI_ISL_3535573, EPI_ISL_2762053, EPI_ISL_2885650, EPI_ISL_2710406, EPI_ISL_3026624, EPI_ISL_3369187, EPI_ISL_2964948, EPI_ISL_3026651, EPI_ISL_2710457, EPI_ISL_2940877, EPI_ISL_3122631, EPI_ISL_3061184, EPI_ISL_2710417, EPI_ISL_2710484, EPI_ISL_3452075, EPI_ISL_3026168, EPI_ISL_2845368, EPI_ISL_2710422, EPI_ISL_2710394, EPI_ISL_3369194, EPI_ISL_2710461, EPI_ISL_2969527, EPI_ISL_2978211, EPI_ISL_2710436, EPI_ISL_3026348, EPI_ISL_2967302, EPI_ISL_2710414, EPI_ISL_2939556, EPI_ISL_2868576, EPI_ISL_2710424, EPI_ISL_2710407, EPI_ISL_3452346, EPI_ISL_3674625, EPI_ISL_2967033, EPI_ISL_2710398, EPI_ISL_3369198, EPI_ISL_2877565, EPI_ISL_3544934, EPI_ISL_2889839, EPI_ISL_3026084, EPI_ISL_3452009, EPI_ISL_2889842, EPI_ISL_2710429, EPI_ISL_2479322, EPI_ISL_3026499, EPI_ISL_2710433, EPI_ISL_2710443, EPI_ISL_3026320, EPI_ISL_2710549, EPI_ISL_3045305, EPI_ISL_2967261, EPI_ISL_3545065, EPI_ISL_2710465, EPI_ISL_2710471, EPI_ISL_2710529, EPI_ISL_3772902, EPI_ISL_2281276, EPI_ISL_3356351, EPI_ISL_2964951, EPI_ISL_2710504, EPI_ISL_2758248, EPI_ISL_3452467, EPI_ISL_2978216, EPI_ISL_2790923, EPI_ISL_2710418, EPI_ISL_2967406, EPI_ISL_2710539, EPI_ISL_3026459, EPI_ISL_3545211, EPI_ISL_2710413, EPI_ISL_2710411, EPI_ISL_2710416, EPI_ISL_2889836, EPI_ISL_3026138, EPI_ISL_2967335, EPI_ISL_3026150, EPI_ISL_3545006, EPI_ISL_2939723, EPI_ISL_3369191, EPI_ISL_3026167, EPI_ISL_2710468, EPI_ISL_2710524, EPI_ISL_3674640, EPI_ISL_2762085, EPI_ISL_3026622, EPI_ISL_3452371, EPI_ISL_2820523, EPI_ISL_2967254, EPI_ISL_3544905, EPI_ISL_3385108, EPI_ISL_3393534, EPI_ISL_2939720, EPI_ISL_3026187, EPI_ISL_2694016, EPI_ISL_2710439, EPI_ISL_3026207, EPI_ISL_3026271, EPI_ISL_2978224, EPI_ISL_2611673, EPI_ISL_2710481, EPI_ISL_2710490, EPI_ISL_2938555, EPI_ISL_3501355, EPI_ISL_2710437, EPI_ISL_2978220, EPI_ISL_3026732, EPI_ISL_3026047, EPI_ISL_2978202, EPI_ISL_2964946, EPI_ISL_2790921, EPI_ISL_3802271, EPI_ISL_2978223, EPI_ISL_3117438, EPI_ISL_2710451, EPI_ISL_3544868, EPI_ISL_3026423, EPI_ISL_2453157, EPI_ISL_2710456, EPI_ISL_2710547, EPI_ISL_2967255, EPI_ISL_3026489, EPI_ISL_2978222, EPI_ISL_3452414, EPI_ISL_2710495, EPI_ISL_2889846, EPI_ISL_3544981, EPI_ISL_2967287, EPI_ISL_3026440, EPI_ISL_2845328, EPI_ISL_2281255, EPI_ISL_3206740, EPI_ISL_3494768, EPI_ISL_2861389, EPI_ISL_3495261, EPI_ISL_2978207, EPI_ISL_3040125, EPI_ISL_3369195, EPI_ISL_3544885, EPI_ISL_2281280, EPI_ISL_2710412, EPI_ISL_2710514, EPI_ISL_2710419, EPI_ISL_2710449, EPI_ISL_3026179, EPI_ISL_2710467, EPI_ISL_3452106, EPI_ISL_2889850, EPI_ISL_2710497, EPI_ISL_2878163, EPI_ISL_2710518, EPI_ISL_2404477, EPI_ISL_3674054, EPI_ISL_2967414, EPI_ISL_2873827, EPI_ISL_2710460, EPI_ISL_3026341, EPI_ISL_2967270, EPI_ISL_2967420, EPI_ISL_2710540, EPI_ISL_2964945, EPI_ISL_3040126, EPI_ISL_3451995, EPI_ISL_2710492, EPI_ISL_3545124, EPI_ISL_2710469, EPI_ISL_2889834, EPI_ISL_2939695, EPI_ISL_3026351, EPI_ISL_2978217, EPI_ISL_3014900, EPI_ISL_2710535, EPI_ISL_3506933, EPI_ISL_2978205, EPI_ISL_2710519, EPI_ISL_3369193, EPI_ISL_2878080, EPI_ISL_2710395, EPI_ISL_2967271, EPI_ISL_2281268, EPI_ISL_2710478, EPI_ISL_2710511, EPI_ISL_2710520, EPI_ISL_2710402, EPI_ISL_3910475, EPI_ISL_3369192, EPI_ISL_1498300, EPI_ISL_2710445, EPI_ISL_3026252, EPI_ISL_3026844, EPI_ISL_2710501, EPI_ISL_3674092, EPI_ISL_2710399, EPI_ISL_2939467, EPI_ISL_2878074, EPI_ISL_2710393, EPI_ISL_3452056, EPI_ISL_2725686, EPI_ISL_3452343, EPI_ISL_3026400, EPI_ISL_2361224, EPI_ISL_2710403, EPI_ISL_2710480, EPI_ISL_2710397, EPI_ISL_2710470, EPI_ISL_2710392, EPI_ISL_2710447, EPI_ISL_2893232, EPI_ISL_2978218, EPI_ISL_2941096, EPI_ISL_2710386, EPI_ISL_2710423, EPI_ISL_2710476, EPI_ISL_2967263, EPI_ISL_2609523, EPI_ISL_2710506, EPI_ISL_3544963, EPI_ISL_2710498, EPI_ISL_3545112, EPI_ISL_3185935, EPI_ISL_2749123, EPI_ISL_2710475, EPI_ISL_2281271, EPI_ISL_3527595, EPI_ISL_2710444, EPI_ISL_2605568, EPI_ISL_2710425, EPI_ISL_2889835, EPI_ISL_2868578, EPI_ISL_3544814, EPI_ISL_3131002, EPI_ISL_3369190, EPI_ISL_3545069, EPI_ISL_3026246, EPI_ISL_2743137, EPI_ISL_3026072, EPI_ISL_3040128, EPI_ISL_2978227, EPI_ISL_3026560, EPI_ISL_3386998, EPI_ISL_2967258, EPI_ISL_3026222, EPI_ISL_2710410, EPI_ISL_2978192, EPI_ISL_3545062, EPI_ISL_2281272, EPI_ISL_2978228, EPI_ISL_2731292, EPI_ISL_3046558, EPI_ISL_3026071, EPI_ISL_2710464, EPI_ISL_2898089, EPI_ISL_3477781, EPI_ISL_2749028, EPI_ISL_2967265, EPI_ISL_2710491, EPI_ISL_2967301, EPI_ISL_2710543, EPI_ISL_2710448, EPI_ISL_2710521, EPI_ISL_3910516, EPI_ISL_2889843, EPI_ISL_2889833, EPI_ISL_2710517, EPI_ISL_2710454, EPI_ISL_2710446, EPI_ISL_2710541, EPI_ISL_2710459, EPI_ISL_2833345, EPI_ISL_2710388, EPI_ISL_3026099, EPI_ISL_2889840, EPI_ISL_2710438, EPI_ISL_3086918, EPI_ISL_2710507, EPI_ISL_2978234, EPI_ISL_3545067, EPI_ISL_2939161, EPI_ISL_2967411, EPI_ISL_2710494, EPI_ISL_2710512, EPI_ISL_2710546, EPI_ISL_2710477, EPI_ISL_3026105, EPI_ISL_2978225, EPI_ISL_2710525, EPI_ISL_2939721, EPI_ISL_2710537, EPI_ISL_2964953, EPI_ISL_3910472, EPI_ISL_2940897, EPI_ISL_3573589, EPI_ISL_2889852, EPI_ISL_3040127, EPI_ISL_3153377, EPI_ISL_2710384, EPI_ISL_2967415, EPI_ISL_2978210, EPI_ISL_2967257, EPI_ISL_2983947, EPI_ISL_3947457, EPI_ISL_2834777, EPI_ISL_2930955, EPI_ISL_3026230, EPI_ISL_3026349, EPI_ISL_2710404, EPI_ISL_3026354,

EPI_ISL_2476304, EPI_ISL_2864234, EPI_ISL_3452063, EPI_ISL_2710405, EPI_ISL_2455323, EPI_ISL_3026322, EPI_ISL_3026065, EPI_ISL_3026085, EPI_ISL_3369197, EPI_ISL_2281267, EPI_ISL_2983944, EPI_ISL_3026542, EPI_ISL_2710502, EPI_ISL_3026482, EPI_ISL_2710515, EPI_ISL_2281260, EPI_ISL_3369196, EPI_ISL_3040131, EPI_ISL_2710522, EPI_ISL_3392090, EPI_ISL_2754641, EPI_ISL_2710420, EPI_ISL_2967280, EPI_ISL_3674412, EPI_ISL_2710472, EPI_ISL_2889838, EPI_ISL_3452368, EPI_ISL_3147606, EPI_ISL_2710532, EPI_ISL_2710408, EPI_ISL_2964950, EPI_ISL_2710415, EPI_ISL_2710432, EPI_ISL_2964947, EPI_ISL_3026330, EPI_ISL_2710455, EPI_ISL_2710435, EPI_ISL_2710531, EPI_ISL_2710450, EPI_ISL_3117439, EPI_ISL_3086915, EPI_ISL_2889837, EPI_ISL_2710509, EPI_ISL_2940852, EPI_ISL_2710442, EPI_ISL_3451950, EPI_ISL_3452410, EPI_ISL_2710430, EPI_ISL_2978195, EPI_ISL_2710503, EPI_ISL_3026180, EPI_ISL_3026240, EPI_ISL_3545063, EPI_ISL_2710385, EPI_ISL_3026340, EPI_ISL_3145495, EPI_ISL_2710499, EPI_ISL_2281269, EPI_ISL_3061186, EPI_ISL_2726487, EPI_ISL_2710434, EPI_ISL_3452017, EPI_ISL_3369189, EPI_ISL_2967283, EPI_ISL_2889841, EPI_ISL_2939552, EPI_ISL_2710544, EPI_ISL_2918863, EPI_ISL_3026126, EPI_ISL_2710387, EPI_ISL_2710510, EPI_ISL_2769380, EPI_ISL_2710548, EPI_ISL_2710440, EPI_ISL_2710421, EPI_ISL_2762006, EPI_ISL_2939711, EPI_ISL_2978236, EPI_ISL_2710479, EPI_ISL_2333740, EPI_ISL_2710545, EPI_ISL_3026814, EPI_ISL_2710458, EPI_ISL_3545055, EPI_ISL_2710401, EPI_ISL_3369186, EPI_ISL_3910542, EPI_ISL_3026457, EPI_ISL_3452370, EPI_ISL_2710526, EPI_ISL_3545177, EPI_ISL_3135410, EPI_ISL_2791285, EPI_ISL_2978196, EPI_ISL_2710533, EPI_ISL_2825031, EPI_ISL_2710485, EPI_ISL_2978226, EPI_ISL_2842626, EPI_ISL_2967256, EPI_ISL_2710528, EPI_ISL_2939458, EPI_ISL_2835867, EPI_ISL_2710474, EPI_ISL_3026299, EPI_ISL_2710493, EPI_ISL_2938899, EPI_ISL_3026087, EPI_ISL_2710396, EPI_ISL_2878449, EPI_ISL_2710473, EPI_ISL_3674682, EPI_ISL_3115157, EPI_ISL_2889845, EPI_ISL_2710453, EPI_ISL_2933451, EPI_ISL_2710513, EPI_ISL_2710496, EPI_ISL_2710536, EPI_ISL_2710400, EPI_ISL_2967386, EPI_ISL_2710542, EPI_ISL_2710390, EPI_ISL_2710482, EPI_ISL_2710462, EPI_ISL_3369188, EPI_ISL_2832893, EPI_ISL_3674561, EPI_ISL_2710391, EPI_ISL_2967267, EPI_ISL_2710427, EPI_ISL_3026508, EPI_ISL_2710463, EPI_ISL_2723768, EPI_ISL_2710452, EPI_ISL_2710488, EPI_ISL_2889847, EPI_ISL_2918837, EPI_ISL_2710409, EPI_ISL_2842627, EPI_ISL_2878081.

Replication	To ensure convergence to the same posterior distribution both MCMC analyses were run 18 (continent-level) and 16 (country-level) times independently and combined. Beyond this, on account of there not being an experimental/control group split in phylogenetic studies
Randomization	Not relevant to a phylogenetic study, since no explicit splits into experimental/control group exist and all available data are used.
Blinding	Not relevant to a phylogenetic study, since it is impossible to bias the phylogenetic placement of a given sequence and selective inclusion/exclusion of sequences based on visual inspection and prediction which part of the tree they would belong is unfeasible for hundreds of sequences.

Reporting for specific materials, systems and methods

We require information from authors about some types of materials, experimental systems and methods used in many studies. Here, indicate whether each material, system or method listed is relevant to your study. If you are not sure if a list item applies to your research, read the appropriate section before selecting a response.

Materials & experimental systems

n/a	Involved in the study
☒	☐ Antibodies
☒	☐ Eukaryotic cell lines
☒	☐ Palaeontology and archaeology
☒	☐ Animals and other organisms
☒	☐ Human research participants
☒	☐ Clinical data
☒	☐ Dual use research of concern

Methods

n/a	Involved in the study
☒	☐ ChIP-seq
☒	☐ Flow cytometry
☒	☐ MRI-based neuroimaging
