# Supplementary material for: Emergence and spread of SARS-CoV-2 lineage B.1.620 with variant of concern-like mutations and deletions
Source: Nat Commun. 2021 Oct 1;12:5769. doi: 10.1038/s41467-021-26055-8 (PMC8486757; doi:10.1038/s41467-021-26055-8)
Supplement: Supplementary file 4 — Description of Additional Supplementary Files [file 41467_2021_26055_MOESM4_ESM.pdf]

# Description of Additional Supplementary Files

Supplementary Data 1.
GISAID acknowledgment table listing all SARS-CoV-2 genomes used in the study.

Supplementary Data 2.
BEAST v1.10.5 XML files used to conduct continent-level and country-level Bayesian phylogeographic analyses.
Sequences are redacted out of the XMLs, per GISAID's Database Access Agreement.
