# Supplementary material for: Emergence and spread of SARS-CoV-2 lineage B.1.620 with variant of concern-like mutations and deletions
Source: Nat Commun. 2021 Oct 1;12:5769. doi: 10.1038/s41467-021-26055-8 (PMC8486757; doi:10.1038/s41467-021-26055-8)
Supplement: Supplementary file 5 — Supplementary Data 1 [file 41467_2021_26055_MOESM5_ESM.pdf]

We gratefully acknowledge the following Authors from the Originating laboratories responsible for obtaining the specimens, as well as the Submitting laboratories where the genome data were generated and shared via GISAID, on which this research is based.

All Submitters of data may be contacted directly via www.gisaid.org

Authors are sorted alphabetically.

Accession ID	Originating Laboratory	Submitting Laboratory	Authors
EPI_ISL_1208402	ACT Pathology	Schwessinger Lab	Ashley Jones; Benjamin Schwessinger; Craig Kennedy; Karina Kennedy; Kevin Murray; Megan McDonald; Ming-Dao Chia; Robert Lanfear; Robyn N Hall
EPI_ISL_1302829, EPI_ISL_1302986, EPI_ISL_2226704, EPI_ISL_2226705, EPI_ISL_2226720	ADMED Microbiologie	Genomics and Transcriptomics, Philip Morris International	David Bornand; Emmanuel Guedj; Manuel Peitsch; Marie-Lise Tritten; Maxime Berthouzoz; Mehdi Auberson; Nicolas Sierro; Nikolai Ivanov; Reto Lienhard; Rémi Dulize
EPI_ISL_2638158	AULSS 1 Dolomiti	Istituto Zooprofilattico Sperimentale delle Venezie	Adelaide Milani; Alessia Schivo; Alice Fusaro; Ambra Pastori; Annalisa Salviato; Antonia Ricci; Calogero Terregino; Edoardo Giussani; Elisa Palumbo; Erika Giorgia Quaranta; Isabella Monne; Luca Tassoni
EPI_ISL_2604197	AULSS 6 Euganea	Istituto Zooprofilattico Sperimentale delle Venezie	Adelaide Milani; Alessia Schivo; Alice Fusaro; Ambra Pastori; Annalisa Salviato; Antonia Ricci; Calogero Terregino; Edoardo Giussani; Elisa Palumbo; Erika Giorgia Quaranta; Isabella Monne; Luca Tassoni
EPI_ISL_426539	AZ SPHL, Arizona Department of Health Services	TGen North	Darrin Lemmer; Dave Engelthaler; Jolene Bowers; Megan Folkerts
EPI_ISL_1560167, EPI_ISL_2040330, EPI_ISL_2370500, EPI_ISL_2489681	Aegis Sciences Corporation	Centers for Disease Control and Prevention Division of Viral Diseases, Pathogen Discovery	Adrian Paskey; Alec Vest; Benjamin Rambo-Martin; Christopher Gulvick; Clinton R. Paden; Cyndi Clark; Dakota Howard; Darlene Wagner; Dhwani Batra; Dillon Nall; Duncan MacCannell; Ethan Sanders; Holly Houdeshell; Jason Caravas; Kara Moser; Matthew Hardison; Matthew Schmerer; Ola Kvalvaag; Patrick Campbell; Peter W. Cook; Rob Case; Scott Sammons; Shatavia Morrison; Shaun Westlund; Vikramsinha Ghorpade; Yvette Unoarumhi
EPI_ISL_806849, EPI_ISL_1575038	Alaska State Virology Laboratory	Alaska State Virology Laboratory	Devin M. Drown; Elva House; Jack Chen; Lisa Smith; Ph.D.; Stephanie DeRonde
EPI_ISL_2169486, EPI_ISL_2169676	Alberta Precision Labs (APL)	Public Health Agency of Canada (PHAC) National Microbiology Laboratory	Buss; Croxen M; Deo A; Dieu P; E; Ferrato C; Gill K; Khan F; Koleva P; Li V; Lloyd C; Lynch T; Ma R; Murphy S; Pabbaraju K; Shokoples S; Thayer J; Tipples G; Whitehouse M; Wong A; Yu C; Zelyas N
EPI_ISL_1385807	Alfa Diagnostica LLC	ONCOGENE LLC	ONCOGENE LLC
EPI_ISL_1583188	Armed Forces Institute of Pathology (AFIP), Dhaka Cantonment	Genomic Research Lab, BCSIR	Abu Sayeed Mohammad Mahmud; Barna Goswami; Eshrar Osman; Iffat Jahan; Md. Ahasan Habib; Md. Kamrul Islam; Md. Murshed Hasan Sarkar; Md. Saddam Hossain; Md. Salim Khan; Mohammad Mizanur Rahman; Mohammad Mohi Uddin; Mohammad Samir Uzzaman; Shahina Akter; Susane Giti; Tanjina Akhter Banu
EPI_ISL_437451	B.J. Medical College and Civil hospital	Gujarat Biotechnology Research Centre	Akanksha Verma; Amit Kanani; Ankit Hinsu; Apurvasinh Puvar; Bhavesh Modi; Bhavya Jindal; Binita Aring; Chaitanya Joshi; Dinesh Kumar; Dipa Kinariwala; Disha Patel; Gaurishankar Shrimali; Geeta Vaghela; Janvi Raval; Kairavi Joshi; Kamlesh J Upadhyay; Madhvi Joshi; Maharshi Pandya; Monika Gandhi; Neeta Khandelwal; Nidhi Sood; Nitin Savaliya; Pinal Trivedi; Pranay Shah; Pritesh Sabara; R D Dixit; Raghawendra Kumar; Ramesh Pandit; Snehal Bagatharia; Sonia Barve; Tejas Shah; Zuber Saiyed
EPI_ISL_2152460, EPI_ISL_2334020, EPI_ISL_2637863	BIOMNIS EUROFINS IVRY	Department of Virology, Henri Mondor University Hospital, Assistance Publique Hôpitaux de Paris, Université Paris-Est Créteil, INSERM U955	Alexandre Soulier; Christophe Rodriguez; Elisabeth Trawinski; Guillaume Gricourt; Jean-Michel Pawlotsky; Melissa N'Debi; Slim Fourati; Vanessa Demontant
EPI_ISL_1265458	BTKLPP Kelas I Makassar	Eijkman Institute for Molecular Biology, Ministry of Research and Technology/National Agency for Research and Innovation; National Institute of Health Research and Development	Amin Soebandrio; Edison Johar; Frilasita A Yudhaputri; Hana Apsari Pawestri; Hidayat Trimarsanto; Iskandar Adnan; Khin Saw Myint; Lydia V. Panggalo; Safarina G Malik; Slamet; Sukma Oktavianthi; Vivi Setiawaty; Willy Agustine
EPI_ISL_529213	Beijing Institute of Microbiology and Epidemiology	Beijing Institute of Microbiology and Epidemiology	Cui, Y.; Fan; Guo, Y.; Hang; Hou, J.; Li, B.; Mi, Z.; Mu, J.; Qin, E.; Song; Teng; Wu, Y.; Xu, Z.; Yajun.; Yang, R.; Yong, Y.; Yue; Zhang, X.
EPI_ISL_1624880	Berkeley Medical Center	WVU and Marshall University Combined Genomics Core Facilities	James Denvir; Peter Perrotta; Peter Stoilov; Ryan Percifield; Wesley Kimble
EPI_ISL_1756607	Biogroup Bio Lam-LCD Saint-Denis	Department of Virology, Henri Mondor University Hospital, Assistance Publique Hôpitaux de Paris, Université Paris-Est Créteil, INSERM U955	Alexandre Soulier; Christophe Rodriguez; Elisabeth Trawinski; Guillaume Gricourt; Jean-Michel Pawlotsky; Melissa N'Debi; Slim Fourati; Vanessa Demontant
EPI_ISL_1498716	BioneXt Lab	Laboratoire national de sante, Microbiology, Microbial Genomics Platform	Anke Wienecke-Baldacchino; Catherine Ragimbeau; Fatu Djabi; Jessica Tapp; Lise Pignon; Raoul Salmon; Tamir Abdelrahman; Thibault Ferrandon
EPI_ISL_1282092	Bioscientia Labor Wermsdorf	Robert Koch Institute	
EPI_ISL_1971577, EPI_ISL_1971594, EPI_ISL_1971703, EPI_ISL_1971763, EPI_ISL_1971881, EPI_ISL_1972027	Broad Institute Clinical Research Sequencing Platform	Infectious Disease Program, Broad Institute of Harvard and MIT	Adams, G.; B.L.; B.W.; Bauer, M.; Birren; Blumenstiel, B.; Brown, C.; Carter, A.; Chaluvadi, S.; D.J.; DeFelice, M.; DeRuff, K.; Dodge, S.; Gabriel, S.; Gallagher, G.; Gladden-Young, A.; Granger, B.; J.E.; K.J.; Lagerborg, K.; Larkin, K.; Lee, M.; Lemieux; Lennon, N.; Loreth, C.; Madoff, L.; McGovern, S.; Meldrim, J.; Normandin, E.; P.C.; Park; Pearlman, L.; Reilly, S.; Rudy, M.; Sabeti; Siddle; Smole, S.; Tomkins-Tinch, C.; Vicente, G.; and MacInnis
EPI_ISL_1371900	C H DE LA POLYNESIE FRANCAISE	CNR Virus des Infections Respiratoires - France SUD	Antonin Bal; Bruno Lina; Gregory Destras; Gwendolyne Burfin; Hadrien Regue; Laurence Josset; Martine Valette; Quentin Semanas
EPI_ISL_1671822	CH Roubaix	CHU Lille - Laboratoire de Virologie	AIT YAHYA Emilie; ALIDJINOU Enagnon Kazali; BOCKET Laurence; CREPIN Michel; DEMAY Christophe; ENGELMANN Ilka; GEFFROY Sandrine; GUIGON Aurélie; LAMBERT Valérie; LAZREK Mouna; NOBILLIAUX Florian; PREVOST Brigitte; THUILLIER Caroline; TINEZ Claire
EPI_ISL_2152575	CH. CHU DE REIMS	Department of Virology, Henri Mondor University Hospital, Assistance Publique Hôpitaux de Paris, Université Paris-Est Créteil, INSERM U955	Alexandre Soulier; Christophe Rodriguez; Elisabeth Trawinski; Guillaume Gricourt; Jean-Michel Pawlotsky; Melissa N'Debi; Slim Fourati; Vanessa Demontant
EPI_ISL_1757704	CH.INTERCOMMUNAL DE CRETEIL	Department of Virology, Henri Mondor University Hospital, Assistance Publique Hôpitaux de Paris, Université Paris-Est Créteil, INSERM U955	Alexandre Soulier; Christophe Rodriguez; Elisabeth Trawinski; Guillaume Gricourt; Jean-Michel Pawlotsky; Melissa N'Debi; Slim Fourati; Vanessa Demontant
EPI_ISL_2228812	CH.TROYES	Department of Virology, Henri Mondor University Hospital, Assistance Publique Hôpitaux de Paris, Université Paris-Est Créteil, INSERM U955	Alexandre Soulier; Christophe Rodriguez; Elisabeth Trawinski; Guillaume Gricourt; Jean-Michel Pawlotsky; Melissa N'Debi; Slim Fourati; Vanessa Demontant
EPI_ISL_2464447	CHI VILLENEUVE ST GEORGES	Department of Virology, Henri Mondor University Hospital, Assistance Publique Hôpitaux de Paris, Université Paris-Est Créteil, INSERM U955	Alexandre Soulier; Christophe Rodriguez; Elisabeth Trawinski; Guillaume Gricourt; Jean-Michel Pawlotsky; Melissa N'Debi; Slim Fourati; Vanessa Demontant
EPI_ISL_2444375	CHU LILLE	CHU Lille - Laboratoire de Virologie	AIT YAHYA Emilie; ALIDJINOU Enagnon Kazali; BOCKET Laurence; CREPIN Michel; DEMAY Christophe; ENGELMANN Ilka; GEFFROY Sandrine; GUIGON Aurélie; LAMBERT Valérie; LAZREK Mouna; NOBILLIAUX Florian; PREVOST Brigitte; THUILLIER Caroline; TINEZ Claire
EPI_ISL_2193991	CHU Nantes Virology	CH Saint Nazaire	Audrey Rodallec; Berthe-Marie Imbert-Marcille; Celine Bressollette-Bodin; Sonia Sachot; Thomas Drumel
EPI_ISL_2194010, EPI_ISL_2194021, EPI_ISL_2194025, EPI_ISL_2194027, EPI_ISL_2194028	CHU Nantes Virology	CHU Nantes Virology	Audrey Rodallec; Berthe-Marie Imbert-Marcille; Celine Bressollette-Bodin; Thomas Drumel
EPI_ISL_2510090	CHUGA-IBP-laboratoire de Virologie	IBP-laboratoire de virologie	Alban Caporossi; Anne Signori -Schmuck; Anne-Karen Faure; Aurélie Truffot; Benjamin Nemoz; Hugo Jardin; Julien Andréani; Julien Lupo; Léa Ponderand; Pascal Poignard; Patrice Morand; Raphaële Germi; Sylvie Larrat
EPI_ISL_1829052, EPI_ISL_1829167, EPI_ISL_1941698, EPI_ISL_1941702, EPI_ISL_1941813, EPI_ISL_2102139, EPI_ISL_2102142, EPI_ISL_2137387, EPI_ISL_2137390, EPI_ISL_2137391, EPI_ISL_2137400, EPI_ISL_2137411, EPI_ISL_2322956, EPI_ISL_2322972, EPI_ISL_2500528, EPI_ISL_2622110			
see above	CHUV	Laboratory of genomics and metagenomics	Claire Bertelli; Damien Jacot; Gilbert Greub; Sebastien Aeby; Sébastien Aeby; Trestan Pillonel
EPI_ISL_1461009, EPI_ISL_2373927, EPI_ISL_2373932, EPI_ISL_2567523	CNR Virus des Infections Respiratoires - France SUD	CNR Virus des Infections Respiratoires - France SUD	Antonin Bal; Bruno Lina; Gregory Destras; Gwendolyne Burfin; Hadrien Regue; Laurence Josset; Martine Valette; Quentin Semanas
EPI_ISL_1190749, EPI_ISL_1190750, EPI_ISL_1190751, EPI_ISL_1190752, EPI_ISL_1190753, EPI_ISL_1190754, EPI_ISL_1190755, EPI_ISL_1190756, EPI_ISL_1190757, EPI_ISL_1190758, EPI_ISL_1190759, EPI_ISL_1190760, EPI_ISL_1190761, EPI_ISL_1190762, EPI_ISL_1190763, EPI_ISL_1190764, EPI_ISL_1190765, EPI_ISL_1190766, EPI_ISL_1190767, EPI_ISL_1190768			
see above	CREMER(Centre de Rechercherches sur les Maladies Emergentes et Ré-émergentes)	TransVIHMI(Recherches Translationnelles sur le VIH et les Maladies Infectieuses)	Ahidjo Ayouba; Celestin Godwe; Christelle Butel; Dowbiss Meta Djomsi; Eitel Mpoudi Ngole; Eric Delaporte; Esemu Livo; Laetitia Serrano; Marcel Tongo; Marie Amougou; Martin Maidadi Foudi; Martine Peeters; Nicole Vidal; Rodrigue Kamga
EPI_ISL_636980	CS Xai Xai	KRISP, KZN Research Innovation and Sequencing Platform	Giandhari J; Ismael N; Nadia Sitoe; Nedio Mabunda; Paulo Arnaldo; Pillay S; Tegally H; Wilkinson E; de Oliveira T
EPI_ISL_979271	Cadham Provincial laboratory	National Microbiology Laboratory (NML)	Anna Majer; Anneliese Landgraff; CanCOGeN's metadata curation team; Darian Hole; David Alexander; Elsie Grudeski; Gary Van Domselaar; Grace Seo; Jared Bullard; Jennifer Tanner; Kerry Dust; Kirsten Biggar; Madison Chapel; Morag Graham; Natalie Knox; Nathalie Bastien; Paul Van Caeseele; Philip Mabon; Public Health Agency of Canada CanCOGeN team; Rhiannon Huzarewich; Russell Mandes; Shari Tyson; Timothy Booth; Yan Li
EPI_ISL_907108	Cancer Biology Department, National Cancer Institute	Cancer Biology Department, National Cancer Institute	A.A.; A.E.; A.N.; Abouelhoda, M.; Ahmed; Bahnassy; Elhosieny; F.W.; Gad; H.K.; Hafez; Hamdy; M.G.; M.M.; M.S.; O.S.; Sedawy; Soliman; Soliman, L.; Zekri
EPI_ISL_1939289	Center for Laboratory Medicine	Center for Laboratory Medicine	Yannick Gerth
EPI_ISL_1843903,	Centogene; Dr. Bauer Laboratoriums GmbH	Robert Koch Institute	

EPI_ISL_2123141, EPI_ISL_2123177			
EPI_ISL_2688652	Centre Henri Becquerel	Centre Hospitalier Universitaire de Rouen Laboratoire de Virologie	Alice Moisan; Fabienne De Oliveira; Marie Leoz
EPI_ISL_1406653	Centre Hospitalier Universitaire Clermont-Ferrand	CHU Clermont-Ferrand, service de virologie	Bisseux Maxime; Combes Patricia; Henquell Cécile; Mirand Audrey
EPI_ISL_2688650, EPI_ISL_2688653, EPI_ISL_2688663, EPI_ISL_2688698, EPI_ISL_2688759, EPI_ISL_2688760, EPI_ISL_2688761, EPI_ISL_2688762, EPI_ISL_2688785, EPI_ISL_2688786			
see above	Centre Hospitalier Universitaire de Rouen Laboratoire de Virologie	Centre Hospitalier Universitaire de Rouen Laboratoire de Virologie	Alice Moisan; Fabienne De Oliveira; Marie Leoz
EPI_ISL_1790123	Centre Pasteur du Cameroun	Institut Pasteur de Dakar	Carniel Elisabeth; Dia Ndongo; Diagne Moussa Moïse; Diallo Amadou; Diop Mamadou; Faye Ousmane; Loucoubar Cheikh; Ndiaye Ndack; Njouom Richard; Sall Amadou Alpha; Sankhe Safietou
EPI_ISL_2434979, EPI_ISL_2442363	Centre de Recherches Médicales de Lambaréné (CERMEL)	Centre de Recherches Médicales de Lambaréné (CERMEL)	Bertrand Lell and Ayola Akim Adegnika; Georgelin Nguema Ondo; Gédéon Prince Manouana; Jean Bernard Lekana-Douki; Joël-Fleury Djoba Siawaya; Moustapha Nzamba Maloum; Rodrigue Bikangui; Sam O'neilla Oye Bingono; Samira Zoa Assoumou; Srinivas reddy Pallerla; Steffen Borrmann; Thirumalaisamy P. Velavan
EPI_ISL_1233103	Centre for Dengue Research and AICBU, Department of Immunology and Molecular Medicine	Centre for Dengue Research and AICBU, Department of Immunology and Molecular Medicine	Chandima Jeewandara; Deshni Jayathilaka; Dinuka Ariyaratne; Diyanath Ranasinghe; Gathsaurie Neelika Malavige; Laksiri Gomes; Tibutius Thanesh Pramanayagam
EPI_ISL_1638013	Centre for Enzyme Innovation, University of Portsmouth / Translational Research Laboratory, Portsmouth Hospitals NHS Trust	COVID-19 Genomics UK (COG-UK) Consortium	Angela Beckett; Christopher Fearn; Kate Cook; Katie Loveson; Salman Goudarzi; Samuel Robson; Scott Elliott; Sharon Glaysher
EPI_ISL_1381254	Centro de Investigacion Biomedica del Noreste (CIBIN)	Unidad de Genomica Avanzada	Alejandro Sanchez-Flores; Alfredo Herrera-Estrella; Alicia Ocana-Mondragon; Angel Gustavo Salas-Lais; Bernardo Martinez-Miguel; Blanca Taboada; Brenda Irasema Maldonado-Meza; Carla Ivon Herrera-Najera; Carlos F. Arias; Celia Boukadida; Clara Esperanza Santacruz-Tinoco; Concepcion Grajales-Muniz; Consorcio Mexicano de Vigilancia Genomica (CoViGen-Mex). Authors (in alphabetical order): Julio Elias Alvarado-Yaah; Fernando Fontove-Herrera; Francisco Pulido; Gloria Elena Espinoza-Ayala; Gloria Maria Molina-Salinas; Gloria Vazquez; Hector Esteban Paz-Juarez; Hector Montoya-Fuentes; Helen Haydee Fernanda Ramirez-Plascencia; Jorge Ivan Salinal-Nevarez; Jose Antonio Enciso-Moreno; Jose Esteban Munoz-Medina; Jose de Jesus Nunez-Contreras; Juan Bautista Chale-Dzul; Luis Alberto Ochoa-Carrera; Margarita Matias-Florentino; Maria Guadalupe Santiago-Mauricio; Maria Guadalupe de Jesus Mireles-Rivera; Nelly Selem-Mojica; Pavel Isa; Ricardo Grande; Santiago Avila-Rios; Victor Hugo Borja-Aburto
EPI_ISL_1585395	Centro de Investigación Biomédica del Noreste (CIBIN)	Instituto Nacional de Enfermedades Respiratorias (INER): Centro de Investigación en Enfermedades Infecciosas (CIENI)	Alejandro Sanchez-Flores; Alfredo Herrera-Estrella; Alicia Ocaña-Mondragón; Angel Gustavo Salas-Lais; Bernardo Martínez-Miguel; Blanca Taboada; Brenda Irasema Maldonado-Meza; Carla Ivón Herrera-Najera; Carlos F. Arias; Celia Boukadida; Clara Esperanza Santacruz-Tinoco; Concepción Grajales-Muñiz; Consorcio Mexicano de Vigilancia Genómica (CoViGen-Mex). Authors (in alphabetical order): Julio Elias Alvarado-Yaah; Célida Duque Molina; Fernando Fontove-Herrera; Francisco Pulido; Gloria Elena Espinosa-Ayala; Gloria María Molina-Salinas; Gloria Vazquez; Hector Esteban Paz-Juárez; Hector Montoya-Fuentes; Helen Haydee Fernanda Ramírez-Plascencia; José Antonio Enciso-Moreno; José Esteban Muñoz-Medina; José de Jesús Nuñez-Contreras; Juan Bautista Chale-Dzul; Luis Alberto Ochoa-Carrera; Margarita Matías-Florentino; María Guadalupe Santiago-Mauricio; María Guadalupe de Jesús Mireles-Rivera; Nelly Sélem-Mojica; Pavel Isa; Ricardo Grande; Santiago Ávila-Ríos; Victor Eduardo Garcia-Arias; Víctor Hugo Borja-Aburto
EPI_ISL_1590223	Centrālā Laboratorija; Eurofins Genomics Europe Sequencing GmbH	Riga East University Hospital-National Microbiology Reference Laboratory; Eurofins Genomics Europe Sequencing GmbH	Arzu Algulieva; Diāna Dusacka; Dārta Pūpola; Ilva Pole; Jana Osīte; Reinis Vangravs; Reinis Zeltmatis; Sergejs Nikisins; Stella Lapiņa; Ģirts Šķenders
EPI_ISL_1278684	Clalit Health Services Laboratories, Israel	Stern Lab	Stern Lab
EPI_ISL_1968946, EPI_ISL_1968959	Clinical Microbiology, Infection Prevention and Control	Section for Molecular Diagnostics	Björn Hallström; Jonas Björkman
EPI_ISL_1993911, EPI_ISL_2102102	Clinical Virology	Clinical Bacteriology	Adrian Egli; Alfredo Mari; Hans Hirsch; Helena MB Seth-Smith; Julia Bielicki; Karoline Leuzinger; Madlen Stange; Manuel Battegay; Tim Roloff
EPI_ISL_2492427	Cliniques universitaires Saint-Luc	UCLouvain/IREC/MBLG	Benoit Kabamba Mukadi; Jean Ruelle
EPI_ISL_632284	Communicable Disease Laboratory, Public Health Directorate	Communicable Disease Laboratory, Public Health Directorate	AlAbbas, Z.; AlHujairi, Z.; AlTaif, Z.; AlWasti, H.
EPI_ISL_823886	DOHMH Central Harlem	New York City Public Health Laboratory	Jade Wang; et al.
EPI_ISL_907075	Department of Biology, University of Basrah	Department of Biology, University of Basrah	Abu-Ali; H.M. and Al-Badran; I.F.
EPI_ISL_2685881	Department of Clinical Microbiology	GIGA Medical Genomics	Bouchra Boujemla; Cécile Meex; Keith Durkin; Maria Artesi; Marie-Pierre Hayette; Nathalie Renotte; Pierrette Melin; Raphaël Boreux; Sébastien Bontems; Vincent Bours
EPI_ISL_1008370	Department of Laboratory Medicine, Division of Clinical Virology, University of Medicine, Vienna	Bergthaler laboratory, CeMM Research Center for Molecular Medicine of the Austrian Academy of Sciences	Andreas Bergthaler; Anna Schedl; Bekir Erguner; Benedikt Agerer; Christoph Bock; Jan Laine; Lukas Endler; Maelle Le Moing; Martin Senekowitsch; Michael Schuster; Thomas Penz
EPI_ISL_648672	Department of Laboratory Medicine, Tan Tock Seng Hospital	Department of Laboratory Medicine, Tan Tock Seng Hospital	Barkham TMS; Chen YYC; Li C; Lim JX; Maurer-Stroh S; Nagarajan N; Sessions OM; Tang WY; Zair X
EPI_ISL_1027639	Department of Microbiology, National Institute for Public Health of Kosova	Charité Universitätsmedizin Berlin, Institut für Virologie	Barbara Mühlemann; Christian Drosten; Donjeta Hajdari; Julia Schneider; Jörn Beheim-Schwarzbach; Talitha Veith; Terry Jones; Victor M Corman; Xhevat Jakupi; Zana Deva
EPI_ISL_732954	Department of Tropical Parasitology	Laboratory of Recombinant Vaccines	Boguslaw Szewczyk; Kirsi Aaltonen; Lukasz Rabalski; Maciej Grzybek; Maciej Kosinski; Ravi Kant; Tarja Sironen; Teemu Smura
EPI_ISL_1406391	Department of Virology	Department of Virology	Aamer Ikram; Abdul Ahad; Austin Leach; Joel Montgomery; John Klena; Ketan Patel; Massab Umair; Melissa Mobley; Muhammad Salman; Nazish Badar; Sana Tamim; Shannon Whitmer; Zaira Rehman
EPI_ISL_766025, EPI_ISL_2608762	Department of Virology and Immunology, University of Helsinki and Helsinki University Hospital, Huslab Finland	Department of Virology, Faculty of Medicine, University of Helsinki, Helsinki, Finland	Essi Korhonen; Hanna Jarva; Hanna Liimatainen; Hannimari Kallio-Kokko; Harri Kangas; Hussein Alburkat; Jenni Virtanen; Maija Lappalainen; Maija Suvanto; Olli Vapalahti; Pekka Ellonen; Phuoc Truong; Ravi Kant; Sari Hannula; Satu Kurkela; Teemu Smura
EPI_ISL_807156	Deva County Emergency Hospital	National Institute of Infectious Diseases-Prof. Dr. Matei Bals Molecular Diagnostics Laboratory	Andreea Tudor; Corina Casangiu; Dan Otelea; Leontina Banica; Marius Surleac; Petre Milu; Simona Paraschiv
EPI_ISL_2403787	Dianalabs SA	Genesupport	Tanguy ARAUD
EPI_ISL_2361095, EPI_ISL_2361096, EPI_ISL_2361097, EPI_ISL_2361099, EPI_ISL_2361135, EPI_ISL_2361138, EPI_ISL_2361139, EPI_ISL_2361140, EPI_ISL_2361148, EPI_ISL_2361150, EPI_ISL_2361151, EPI_ISL_2361152, EPI_ISL_2361162, EPI_ISL_2361167, EPI_ISL_2361169, EPI_ISL_2361170, EPI_ISL_2361171, EPI_ISL_2361172, EPI_ISL_2361180, EPI_ISL_2361182, EPI_ISL_2361186, EPI_ISL_2361194, EPI_ISL_2361195, EPI_ISL_2361196, EPI_ISL_2361198, EPI_ISL_2361206, EPI_ISL_2361207, EPI_ISL_2361208, EPI_ISL_2361209, EPI_ISL_2361210, EPI_ISL_2361211, EPI_ISL_2361212, EPI_ISL_2361213, EPI_ISL_2361214, EPI_ISL_2361215, EPI_ISL_2361216, EPI_ISL_2361217, EPI_ISL_2361218, EPI_ISL_2361219, EPI_ISL_2361220, EPI_ISL_2361221, EPI_ISL_2361222, EPI_ISL_2361223, EPI_ISL_2361225, EPI_ISL_2361226, EPI_ISL_2361227, EPI_ISL_2361228, EPI_ISL_2361229, EPI_ISL_2361230, EPI_ISL_2361231, EPI_ISL_2361232, EPI_ISL_2361233, EPI_ISL_2361234, EPI_ISL_2361235, EPI_ISL_2361236, EPI_ISL_2361237, EPI_ISL_2361238, EPI_ISL_2361239, EPI_ISL_2361240, EPI_ISL_2361241, EPI_ISL_2361242, EPI_ISL_2361243, EPI_ISL_2361244, EPI_ISL_2361245, EPI_ISL_2361246, EPI_ISL_2361247, EPI_ISL_2361248, EPI_ISL_2361249, EPI_ISL_2361250, EPI_ISL_2361251, EPI_ISL_2361252, EPI_ISL_2361253, EPI_ISL_2361254, EPI_ISL_2361255, EPI_ISL_2361256, EPI_ISL_2361257, EPI_ISL_2361258, EPI_ISL_2361259, EPI_ISL_2361260, EPI_ISL_2361261, EPI_ISL_2361262, EPI_ISL_2361263, EPI_ISL_2361264, EPI_ISL_2361265			
see above	Division of Emerging Infectious Diseases, Bureau of Infectious Diseases Diagnosis Control, Korea Disease Control and Prevention Agency	Division of Emerging Infectious Diseases, Bureau of Infectious Diseases Diagnosis Control, Korea Disease Control and Prevention Agency	Ae Kyung Park; Chae Young Lee; Eun-Jin Kim; Heui Man Kim; Il-Hwan Kim; Jeong-Ah Kim; Jeong-Min Kim
EPI_ISL_1502178	Division of Medical Virology, National Health Laboratory Service (NHLS), Tygerberg Hospital / Stellenbosch University	Division of Medical Virology, Stellenbosch University and NHLS Tygerberg Hospital	Bronwyn Kleinhans; Gert van Zyl; Susan Engelbrecht; Wolfgang Preiser
EPI_ISL_1014570, EPI_ISL_1014617, EPI_ISL_1456512, EPI_ISL_1522216	Dutch COVID-19 response team	National Institute for Public Health and the Environment (RIVM)	Adam Meijer; AnneMarie van den Brandt; Annelies Kroneman; Bas van der Veer; Chantal Reusken; Dennis Schmitz; Dirk Eggink; Euníce Then; Florian Zwagemaker; Harry Vennema; James Groot; Jeroen Cremer; Jolienke Hardeman; Karim Hajji; Kim Freriks; Linda van de Nes; Lisa Wijsman; Lynn Aarts; Melissa van Tuil; Robert Kohl; Ryanne Jaarsma; Sanne Bos; Sharon van den Brink; Sjoerd Kuiling; on behalf of the national COVID-19 response team
EPI_ISL_2102132, EPI_ISL_2686828	EHNV	Laboratory of genomics and metagenomics	Claire Bertelli; Damien Jacot; Gilbert Greub; Sébastien Aeby; Trestan Pillonel
EPI_ISL_1571290, EPI_ISL_1643902, EPI_ISL_1643915, EPI_ISL_1845845, EPI_ISL_1845921, EPI_ISL_1845926, EPI_ISL_2110753, EPI_ISL_2123767, EPI_ISL_2315648, EPI_ISL_2316829			
see above	Eurofins LifeCodexx GmbH	Robert Koch Institute	
EPI_ISL_912369	Fondation Congolaise pour la recherche medicale (FCRM), Francine Ntoumi	NGS Competence Center Tuebingen, Institut für Medizinische Mikrobiologie und Hygiene, Universitaetsklinikum Tübingen	Angel Angelov
EPI_ISL_1919296	Furst Medical Laboratory	Norwegian Institute of Public Health, Department of Virology	Atiya R Ali; Debech Nadia; Engebretsen Serina Beate; Garcia Llorente Ignacio; Hilde Elshaug; Hilde Vollan; Jon Bråte; Kamilla Heddeland Instefjord; Karoline Bragstad; Kathrine Stene-Johansen; Marie Paulsen Madsen; Olav Hungnes; Pedersen Benedikte Nevjen; Rasmus Riis Kopperud
EPI_ISL_1239005	GA Department of Public Health	GA Department of Public Health	Aliyah Fields; Cynthia Dixey; Jonathan Edwards; Stacy Reeves; Taylor Smith; Tonia Parrott
EPI_ISL_2152589	GH A.CHENEVIER-H.MONDOR	Department of Virology, Henri Mondor University Hospital, Assistance Publique Hôpitaux de Paris, Université Paris-Est Créteil, INSERM U955	Alexandre Soulier; Christophe Rodriguez; Elisabeth Trawinski; Guillaume Gricourt; Jean-Michel Pawlotsky; Melissa N'Debi; Slim Fourati; Vanessa Demontant
EPI_ISL_1915592	GHDC - SITE ST JOSEPH	Institut de Pathologie et Genetique (IPG)	Jérémie Gras; Pascale Hilbert
EPI_ISL_406798	General Hospital of Central Theater Command of People's Liberation Army of China	BGI & Institute of Microbiology, Chinese Academy of Sciences & Shandong First Medical University & Shandong Academy of Medical Sciences & General Hospital of Central Theater Command of People's Liberation Army of China	Weifeng Shi and Zhenhong Hu; Weijun Chen; Yuhai Bi
EPI_ISL_746484, EPI_ISL_1167764, EPI_ISL_1167777	Genetica Molecular and Subdepartamento de Virologia ISP Chile	Instituto de Salud Publica de Chile	Andres Castillo; Barbara Parra; Gisselle Barra; Jaime Lagos; Javier Tognarelli; Jorge Fernandez; Karen Orostica; Loredana Arata; Patricia Bustos; Rodrigo Fasce
EPI_ISL_1595851	Genome Analysis Center, Yamanashi Central Hospital	Genome Analysis Center, Yamanashi Central Hospital	Yosuke Hirotsu

EPI_ISL_496790, EPI_ISL_1502988	Gorgas Memorial Laboratory of Health Studies	Gorgas Memorial Laboratory of Health Studies	Alexander A Martinez; Castillo Jorge; Claudia Gonzalez Sandra Lopez-Verges; Danilo Franco; Franco Danilo; Gonzalez Claudia; Jessica Gondola; Leyda Abrego; Lopez-Verges Sandra; Marlenne Castillo; Martinez Alexander; Moreno Ambar; Moreno Brechla; Oris Chavarria; Ortiz Alma
EPI_ISL_2535955	Gravity Diagnostics, LLC	Gravity Diagnostics, LLC	Gravity Diagnostics
EPI_ISL_2322955	HIB	Laboratory of genomics and metagenomics	Claire Bertelli; Damien Jacot; Gilbert Greub; Sébastien Aeby; Trestan Pillonel
EPI_ISL_2651126	HOME QUARANTINE TASKFORCE	Hong Kong Department of Health	Alan K.L. Tsang; Dominic N.C. Tsang; Edman T.K. Lam; Ken H.L. Ng; Peter C.W. Yip; Rickjason C.W. Chan
EPI_ISL_2010575	Helix/Illumina	Centers for Disease Control and Prevention Division of Viral Diseases, Pathogen Discovery	Adrian Paskey; Alexandre Bolze; Ary Ascencio; Benjamin Rambo-Martin; Brad Sickler; Charlotte Rivera-Garcia; Christine Tran; Christopher Gulvick; Clinton R. Paden; Dakota Howard; Darlene Wagner; David Becker; Dhwani Batra; Duncan MacCannell; Efren Sandoval; Eileen de Feo; Elizabeth Cirulli; Eric Allen; Geraint Levan; James Lu; Jan Antico; Jason Caravas; Jason Nguyen; Jimmy Ramirez; Jingtao Liu; Kara Moser; Kelly Schiabor Barrett; Kim Gietzen; Magnus Isaksson; Marc Laurent; Matthew Schmerer; Matthew Tolentino; Nicole L. Washington; Peter W. Cook; Phil Febbo; Ryan Cho; Scott Sammons; Shannon Wickline; Shatavia Morrison; Sherry Wang; Simon White; Tyler Cassens; William Lee; Yvette Unoarumhi
EPI_ISL_767874	Histopath	NSW Health Pathology - Institute of Clinical Pathology and Medical Research; Westmead Hospital; University of Sydney	CIDM-PH et al.
EPI_ISL_1582678, EPI_ISL_1675082, EPI_ISL_1675084, EPI_ISL_1700651, EPI_ISL_1789037, EPI_ISL_1789089, EPI_ISL_1789090, EPI_ISL_1789091, EPI_ISL_1789092, EPI_ISL_1789093, EPI_ISL_1789094, EPI_ISL_1789095, EPI_ISL_1789096, EPI_ISL_1789097, EPI_ISL_1789100, EPI_ISL_1821604, EPI_ISL_1915140, EPI_ISL_1915177, EPI_ISL_2029977, EPI_ISL_2188340, EPI_ISL_2188415, EPI_ISL_2188468, EPI_ISL_2293321, EPI_ISL_2293322, EPI_ISL_2293323, EPI_ISL_2293498, EPI_ISL_2374183, EPI_ISL_2374411, EPI_ISL_2464024, EPI_ISL_2531831, EPI_ISL_2532297			
see above	Hospital	National Reference Center for Viruses of Respiratory Infections, Institut Pasteur, Paris	Adrien Pain; Amaury Vaysse; Angela Brisebarre; Camille Capel; Ch De Cornouaille; Christophe Malabat; CléMence Guillaume; Corinne Maufrais; CéLine Bressollette; Damien Mornico; Emmanuelle Permal; Etienne Simon-Lorière; Farida Moreau Benaoudia; Frédéric Lemoine; Gael Millot; JéRome Guinard; Louise Lefrançois; Marion Barbet; Maud Vanpeene; Méline Bizard; Nabil Gastli; Pascale Martres; Pierre Lechat; Sandrine Castelain; Sylvie Behillil; Sylvie Van der Werf; Sylvie van der Werf; Thibault Guinoiseau; Victoire Baillet; Vincent Enouf
EPI_ISL_1120614	Hospital Margarita Maza de Juarez	CIAD LDM-LGM	Bruno Gome-Gil; Julissa Enciso-Ibarra
EPI_ISL_1963950, EPI_ISL_1963963, EPI_ISL_1963964, EPI_ISL_1963980, EPI_ISL_1963982, EPI_ISL_1963994, EPI_ISL_1964008, EPI_ISL_1964016, EPI_ISL_1964053, EPI_ISL_1964056, EPI_ISL_1964065, EPI_ISL_1964077			
see above	Hospital Universitari Bellvitge	Microbiology Department	Aida Gonzalez-Diaz; Carmen Ardanuy; Jordi Camara; Jordi Niubó; Laura Calatayud; M Angeles Domínguez; Miguel Fernandez-Huerta; Sara Marti
EPI_ISL_2467767	Hôpital Bichat Claude Bernard, Laboratoire de Virologie	IAME UMR1137 Inserm, Université de Paris, Hôpital Bichat	Alexandre Storto; Amélie Recoing; Antoine Bridier-Nahmias; Benoit Visseaux; Charlotte Charpentier; Diane Descamps; Gilles Collin; Lena Daniel; Mélanie Bertine; Nadhira Houhou-Fidouh; Quentin Le Hingrat; Siham Hamri
EPI_ISL_2228884, EPI_ISL_2333768	Hôpital Cochin	Department of Virology, Henri Mondor University Hospital, Assistance Publique Hôpitaux de Paris, Université Paris-Est Créteil, INSERM U955	Alexandre Soulier; Christophe Rodriguez; Elisabeth Trawinski; Guillaume Gricourt; Jean-Michel Pawlotsky; Melissa N'Debi; Slim Fourati; Vanessa Demontant
EPI_ISL_1755103, EPI_ISL_1757775	Hôpital Necker-Enfants malades	Department of Virology, Henri Mondor University Hospital, Assistance Publique Hôpitaux de Paris, Université Paris-Est Créteil, INSERM U955	Alexandre Soulier; Christophe Rodriguez; Elisabeth Trawinski; Guillaume Gricourt; Jean-Michel Pawlotsky; Melissa N'Debi; Slim Fourati; Vanessa Demontant
EPI_ISL_2637798	Hôpital Paul Brousse	Department of Virology, Henri Mondor University Hospital, Assistance Publique Hôpitaux de Paris, Université Paris-Est Créteil, INSERM U955	Alexandre Soulier; Christophe Rodriguez; Elisabeth Trawinski; Guillaume Gricourt; Jean-Michel Pawlotsky; Melissa N'Debi; Slim Fourati; Vanessa Demontant
EPI_ISL_1381065	IAL Regional de Santo Andre	Instituto Adolfo Lutz, Interdiciplinary Procedures Center, Strategic Laboratory	Caio Vinicius Dias Lopes; Claudia Regina Gonçalves; Claudio Tavares Sacchi; Erica Valessa Ramos Gomes; Karoline Rodrigues Campos
EPI_ISL_1577798	INHRR	Laboratorio de Virología Molecular	Aguilar M; Alarcon V; D Angelo P; Delgado M; Garzaro D; Jaspe RC; Loureiro CL; Pujol FH; Rangel HR; Rodriguez L; Zambrano JL
EPI_ISL_1419722	INSACOG-WB	National Institute of Biomedical Genomics – INSACOG	Ajay Chakraborti; Arindam Maitra; Bhaswati Bandyopadhyay; Nidhan Kumar Biswas; Saumitra Das; Sreedhar Chinnaswamy; Tamal Ghosh
EPI_ISL_1517423	Inciensa, Instituto Costarricense de Investigación y Enseñanza en Nutrición y Salud	Inciensa, Instituto Costarricense de Investigación y Enseñanza en Nutrición y Salud	Cristian Peréz-Corrales; Valeria Peralta-Barquero & Gallegos-Carrillo B
EPI_ISL_1587703, EPI_ISL_1733152, EPI_ISL_2014315, EPI_ISL_2014756, EPI_ISL_2087544, EPI_ISL_2187220, EPI_ISL_2283507			
see above	Infinity Biologix	Centers for Disease Control and Prevention Division of Viral Diseases, Pathogen Discovery	Adrian Paskey; Benjamin Rambo-Martin; Chirayu Goswami; Christian Bixby; Christopher Gulvick; Clinton R. Paden; Dakota Howard; Darlene Wagner; Dhwani Batra; Duncan MacCannell; Jason Caravas; Jonathan Schultz; Kara Moser; Matthew Schmerer; Peter W. Cook; Robin Grimwood; Russ Hager; Scott Sammons; Shatavia Morrison; Yihe Wang; Yvette Unoarumhi
EPI_ISL_1509002	Institut National d'hygiène	Unité Mixte Internationale TransVIHMI (UMI 233 IRD – U1175 INSERM - Université de Montpellier) IRD (Institut de recherche pour le développement)	Abla A. KONOU; Adodo SADJI; Ahidjo AYOUBA; Akoélé SILIADIN; Alassane OURO-MEDELI; Amivi EHLAN; Améyo DORKENOO; Anoumou DAGNRA; Christelle BUTEL; Déléma MABA; Eric DELAPORTE; Issaka Maman; Kokou TEGUENI; Koku AGBODEKA; Laetitia SERRANO; Martine PEETERS; Messanh DOUFFAN; Mireille PRINCE-DAVID; Mounerou SALOU; Sidonie A.M.KAGNISSODE; Sika DOSSIM; Syntyche DEVATCHAGNI; Wembo A. HALATOKO
EPI_ISL_999032	Institut Pasteur de Guinée	Institut Pasteur de Dakar	Dia Ndongo; Diagne Moussa Moïse; Diallo Amadou; Diop Mamadou; Faye Ousmane; Grayo Solene; Loucoubar Cheikh; Mbengue Safietou Sankhe; Ndiaye Ndack; Sall Amadou Alpha; Tordo Noel
EPI_ISL_2262447	Institut für Klinische Transfusionsmedizin und Immungenetik DRK Blutspendedienst Baden-Württemberg	Robert Koch Institute	
EPI_ISL_1811302	Institute for Infectious Diseases	Institute for Infectious Diseases	Alban Ramette; Christian Baumann; Cora Sägesser; Franziska Suter-Riniker; Miguel A Terrazos Miani; Pascal Bittel; Peter Keller; Stefan Neuenschwander; Stephen L Leib
EPI_ISL_2153441	Institute for Infectious Diseases, University of Bern	Institute for Infectious Diseases, University of Bern	Alban Ramette; Christian Baumann; Cora Sägesser; Franziska Suter-Riniker; Miguel A Terrazos Miani; Pascal Bittel; Peter Keller; Stefan Neuenschwander; Stephen L Leib
EPI_ISL_1941537	Institute of Medical Microbiology and Hospital Hygiene	Institute of Medical Microbiology and Hospital Hygiene	Aljoscha Tersteegen; Prof. Dr. Achim Kaasch
EPI_ISL_697791, EPI_ISL_728204	Institute of Microbiology, Universidad San Francisco de Quito	Institute of Microbiology, Universidad San Francisco de Quito	Belén Prado-Vivar; Bernardo Gutiérrez; Gabriel Trueba; Jonathan Araujo; Juan José Guadalupe; Michelle Grunauer; Monica Becerra-Wong; Patricio Reyes; Patricio Rojas-Silva; Paúl Cárdenas; Sully Márquez; Tania Guayasamin; Verónica Barragán
EPI_ISL_1443661	Institute of Microbiology, Universidad San Francisco de Quito	Omics Sciences Laboratory	; Andrea Cunguan; Belén Prado-Vivar; Bernardo Gutiérrez; Darlyn Amaya; Dayron Brossad; Derly Andrade Molina; Emily Sulay Saltos Montalvo; Fernanda Zurita; Gabriel Morey León; Gabriel Trueba; Juan Carlos Fernández Cadena; Juan José Guadalupe; Katheryn Sacheri Viteri; Michelle Grunauer; Monica Becerra-Wong; Nabih Dahik; Patricio Rojas-Silva; Paula Juliana Gavilanes Jarrín; Paúl Cárdenas; Rubén Armas González; Sully Márquez; Verónica Barragán
EPI_ISL_1675656	Institute of Molecular and Translational Medicine / Laboratory of Experimental Medicine, Faculty of Medicine and Dentistry, Palacky University and University Hospital Olomouc	Institute of Molecular and Translational Medicine / Laboratory of Experimental Medicine, Faculty of Medicine and Dentistry, Palacky University	Barbora Blumová; Hana Jaworek; Marián Hajdúch; Rastislav Slavkovský; Tomáš Pospíšil; Vladimíra Koudeláková
EPI_ISL_577741, EPI_ISL_875538	Institute of Virology, Biomedical Research Center of the Slovak Academy of Sciences, Bratislava	Faculty of Natural Sciences, Comenius University, Bratislava	Boris Klempa; Broňa Brejová; Dominika Fričová; Edita Staroňová; Elena Tichá; Jozef Nosek; Juraj Kopáček; Kristína Boršová; Martina Ličková; Martina Neboháčová; Monika Sláviková; Sabina Fumačová Havlíková; Tomáš Vinař; Viktória Hodorová; Viktória Čabanová; Ľubomíra Lukáčiková
EPI_ISL_491951	Instituto Nacional de Investigación en Salud Pública - INSPI	INSPI - Charité	Alberto Orlando; Alexandra Usiña; Alfredo Bruno Caicedo; Andres Moreira-Soto; Anna-Lena Sander; Denisses Portugal; Domenica de Mora Coloma; Jan Felix Drexler; Juan Carlos Zeballos; Manuel Gonzalez; Maritza Olmedo; Nina Krause; Silvia Salgado
EPI_ISL_1545316, EPI_ISL_1545320	Instituto Nacional de Investigação em Saúde	KRISP, KZN Research Innovation and Sequencing Platform	Afonso P; David K; Emmanuel SJ; Freitas RH; Giandhari J; Inglês L; Lutucuta S; Miranda J; Morais J; Mufinda M; Naidoo Y; Neto Z; Paulo A Carralero RR Paixão JP; Pereira A; Pillay S; Tegally H; Wilkinson E; de Oliveira T
EPI_ISL_536504	Instituto Nacional de Salud	Laboratorio de Infecciones Respiratorias Agudas	David Tarazona; Dennis Carhuaricra; Eduardo Juscamayta Lopez; Faviola Valdivia Guerrero; Lenin Maturrano Hernandez; Nancy Rojas Serrano; Ronnie Gavilan Chavez
EPI_ISL_1303367	Instituto Nacional de Salud- Dirección de Redes de Laboratorios de Salud Pública	Instituto Nacional de Salud- Dirección de Investigación en Salud Pública	Carlos Franco-Muñoz; Carmen Osorio; Diana Malo; Diego A. Álvarez-Díaz; Diego Andrés Prada; Gerardo Santamaría; Hector Alejandro Ruiz-Moreno; Jhonnatan Reales-González; Juan Camilo Martinez; Julian Naizaque; Katherine Laiton-Donato; Lisseth Pardo; Magdalena Wiesner; Marcela Mercado-Reyes; Maria T. Herrera-Sepúlveda; Marta Lopez Blanco; Martha Lucia Ospina Martinez; Sergio Gomez; Sheryll Corchuelo; Ángela Alarcon Cruz
EPI_ISL_887486	Instituto Nacional de Saude (INS), Mozambique	KRISP, KZN Research Innovation and Sequencing Platform	Giandhari J; Nadia Sitoe; Nalia Ismael; Nedio Mabunda; Paulo Arnaldo; Pillay S; Tegally H; Wilkinson E; de Oliveira T
EPI_ISL_1117135	Instituto Nacional de Saude (INSA)	Instituto Nacional de Saude (INSA)	Borges et al
EPI_ISL_1854262	Instituto Nacional de Saude (INSA) and Institute of Biomedicine (iBiMed), Universidade de Aveiro	Instituto Nacional de Saude (INSA) and Institute of Biomedicine (iBiMed), Universidade de Aveiro	Borges et al
EPI_ISL_1168493, EPI_ISL_1168616	Instituto de Diagnostico y Referencia Epidemiologicos INDRE_RNLSP	Instituto de Diagnostico y Referencia Epidemiologicos (INDRE)	Abril Rodriguez-Maldonado; Ariadna Medina-Benitez; Claudia Wong-Arambula; David Fragoso-Fonseca; Ernesto Ramirez-Gonzalez.; Gisela Barrera-Badillo; Irma Lopez-Martinez; Joaquin Quiroz-Mercado; Lucia Hernandez-Rivas; Natividad Cruz-Ortiz; Sergio Rangel-Guerrero; Tatiana Nunez-Garcia; Vanessa Rivero-Arredondo
EPI_ISL_2131284	Instituut Tropische Geneeskunde	Labo Klinische Biologie, UZA	Basil Britto Xavier; Christine Lammens; Herman Goossens; Jasmine Coppens; Marie Le Mercier; Veerle Matheeussen
EPI_ISL_1167145	Iressef Genomics lab	L'institut de Recherche en Santé, de Surveillance Épidémiologique et de Formation (IRESSEF)	Abdou PADANE; Abdoulie KANTEH; Abdul Karim SESAY; Ambroise AHOUIDI; Aminata DIA; Aminata MBOUP; Astou Gaye GAYE; Barada CISSE; Birahim Piere NDIAYE; Gora LO; Khadim GUEYE; Moustapha MBOW; Nafisatou LEYE; Ndeye Coumba Toure KANE; Papa Alassane DIAW; Souleymane MBOUP; Yacine DIA
EPI_ISL_1608021	Jessa	Jessa	Cruys et al. on behalf of the Jessa_cmdLab
EPI_ISL_1805832, EPI_ISL_1973436, EPI_ISL_1973437	Johns Hopkins Hospital Department of Pathology	Johns Hopkins Hospital Department of Pathology	Adannaya Amadi; C. Paul Morris; Chun Huai Luo; Heba H. Mostafa; Matthew Schwartz; Nicholas Gallagher
EPI_ISL_1440264	KEMRI-Wellcome Trust Research Programme,Kilifi	KEMRI-Wellcome Trust Research Programme,Kilifi	Githinji G.; Mburu M.W.; Mohamed K.S.; deLaurent Z.
EPI_ISL_968849, EPI_ISL_968873	KEMRI-Wellcome Trust Research Programme/KEMRI-CGMR-C Kilifi	KEMRI-Wellcome Trust Research Programme/KEMRI-CGMR-C Kilifi	Githinji et al
EPI_ISL_1382294,	KU Leuven, Rega Institute, Clinical and	KU Leuven, Rega Institute, Clinical and Epidemiological Virology	Bert Vanmechelen; Joan Marti-Carerras; Piet Maes; Tony Wawina-Bokalanga

EPI_ISL_2424358	Epidemiological Virology		
EPI_ISL_2534007	Kaiser Permanente Washington Health Research Institute	Genomics and Discovery, Respiratory Viruses Branch, Division of Viral Diseases, Centers for Disease Control and Prevention	Adam Retchless; Anna Kelleher; Anna Uehara; Brian Lynch; Clinton R. Paden; Dhwani Batra; Haibin Wang; Han Jia Justin Ng; Jasmine Padilla; Jing Zhang; Justin Lee; Krista Queen; Mark Burroughs; Mili Sheth; Morgan Davis; Peter Cook; Rachel Marine; Sarah Nobles; Suxiang Tong; Tara Coalter; Yan Li; Ying Tao
EPI_ISL_2689476	Kath. Marienkrankenhaus	Heinrich Pette Institute, Leibniz Institute for Experimental Virology	Adam Grundhoff; Alexis Robitaille; Johannes Knobloch; Martin Aepfelbacher; Nicole Fischer; Thomas Günther
EPI_ISL_2095894	Klaipedos universitetine ligonine	National Public Health Surveillance Laboratory	Ana Steponkiene; Danas Baksa; Jelena Razmuk; Lukas Vasionis; Lukas Zemaitis; Migle Gabrielaite; Svajune Muralyte
EPI_ISL_1008420	Klinisk mikrobiologi	The Public Health Agency of Sweden	Anna Risberg; Anna-Malin Linde; Carlo Berg; Karin Tegmark-Wisell; Maria Lind Karlberg; Mattias Haukland; Mia Brytting; Noura Walai; Oskar Karlsson Lindsjo; Petra Edquist; Petra Holmstrom; Reza Advani; Sofia Stamouli
EPI_ISL_2278470	LABO BIOMEDICA	CHU Purpan - Laboratoire de Virologie - Institut Fédératif de Biologie	Bulach T.; Donnadieu C.; Izopet J.; Latour J.; Milhes M.; Nicot F.; Ranger N.; Salin G.; Tremeaux P.
EPI_ISL_2482791	LABORATOIRE CERBALLIANCE PLT VILLON	CNR Virus des Infections Respiratoires - France SUD	Antonin Bal; Bruno Lina; Gregory Destras; Gwendolyne Burfin; Hadrien Regue; Laurence Josset; Martine Valette; Quentin Semanas
EPI_ISL_2629288	LABORATOIRE CREAVALLEE	CNR Virus des Infections Respiratoires - France SUD	Antonin Bal; Bruno Lina; Gregory Destras; Gwendolyne Burfin; Hadrien Regue; Laurence Josset; Martine Valette; Quentin Semanas
EPI_ISL_1483032	LESP Nuevo Leon	Instituto de Diagnostico y Referencia Epidemiologicos (INDRE)	Abril Rodriguez-Maldonado; Ariadna Medina-Benitez; Claudia Wong-Arambula; Ernesto Ramirez-Gonzalez.; Gisela Barrera-Badillo; Irma Lopez-Martinez; Joaquin Quiroz-Mercado; Lucia Hernandez-Rivas; Natividad Cruz-Ortiz; Sergio Rangel-Guerrero; Tatiana Nunez-Garcia; Vanessa Rivero-Arredondo
EPI_ISL_1688635, EPI_ISL_1688660, EPI_ISL_1827069, EPI_ISL_1827401, EPI_ISL_2191990, EPI_ISL_2192008, EPI_ISL_2192010, EPI_ISL_2192021, EPI_ISL_2192037, EPI_ISL_2192125			
see above	Lab voor klinische biologie	Lab voor klinische biologie	Bruno Verhasselt; Hannelore Hamerlinck; Marija Janevska
EPI_ISL_2265552	LabKom - MVZ Labor Bochum MLB GmbH	Robert Koch Institute	
EPI_ISL_456382, EPI_ISL_1967901	LabPLUS	Institute of Environmental Science and Research (ESR)	Anja Werno; Antje van der Linden; Arlo Upton; Chris Mansell; David Hammer; Dragana Drinkovic; Erasmus Smit; Gary McAuliffe; Hana Sofia Andersson; Hermes Perez; James Ussher; Jill Sherwood; Jing Wang; Joep de Ligt; Josh Freeman; Julia Howard; Juliet Elvy; Lauren Jelly; Mary DeAlmeida; Matt Blakiston; Matt Storey; Matthew Rogers; Max Bloomfield; Michael Addidle; Michelle Balm; Muhammad Faisal; Nikki Freed; Olin Silander; Olivia Stroeven; Rachel Boyle; Sally Roberts; SallyAnn Harbison; Sarah Jefferies; Sharmini Muttaiyah; Susan Morpeth; Susan Taylor; Timothy Blackmore; Vani Sathyendran; Veronica Playle; Virginia Hope; Xiaoyun Ren
EPI_ISL_1675013, EPI_ISL_1675021, EPI_ISL_2029615, EPI_ISL_2029621, EPI_ISL_2029629, EPI_ISL_2029714, EPI_ISL_2029742, EPI_ISL_2029787, EPI_ISL_2029888, EPI_ISL_2029936, EPI_ISL_2030060, EPI_ISL_2293601, EPI_ISL_2464209, EPI_ISL_2612587, EPI_ISL_2627977			
see above	Labo Analyses Med	National Reference Center for Viruses of Respiratory Infections, Institut Pasteur, Paris	Adrien Pain; Amaury Vaysse; Angela Brisebarre; Anne Holstein; Brieuc Gestin; Camille Capel; Christophe Malabat; Corinne Maufrais; Edouard Lamandin; Emmanuelle Permal; Etienne Simon-Lorière; Fabienne Artur; Frédéric Lemoine; Jean-Christophe Denis; Karine Breant; Louise Lefrançois; Marion Barbet; Maud Vanpeene; Melanie Caron; Méline Bizard; Pascale Martres; Pierre Lechat; Pierre-Yves Leonard; StéPhane Romand; Sylvie Behillil; Sylvie Van der Werf; Vincent Enouf
EPI_ISL_1495980	Laboratoire Biorylis	National Reference Center for Viruses of Respiratory Infections, Institut Pasteur, Paris	Angela Brisebarre; Camille Capel; Etienne Simon-Lorière; Louise Lefrançois; Marion Barbet; Maud Vanpeene; Méline Bizard; Potiron Grégoire; Sylvie Behillil; Sylvie van der Werf; Vincent Enouf
EPI_ISL_2373973	Laboratoire Virologie Saint Louis APHP	Laboratoire Virologie Saint Louis APHP	Constance Delaugerre; Jérome Le Goff; Linda Feghoul; Marie Laure Chaix; Marie Laure Néré; Maud Salmona; Severine Mercier Delarue; Sophia Achaibou
EPI_ISL_1137621	Laboratoire central de Virologie	Laboratoire de Biotechnologie	Abdelomunim Essabbar; Amal Zouaki; Ghizlane EL Amin; Hakima Kabbaj; Lahcen Belyamani and Azeddine Ibrahimi; Mouna Ouadghiri; Myriam Seffar; Saaïd Amzazi; Tarik Aanniz
EPI_ISL_660464	Laboratoire de Microbiologie CHU Souro Sanou	Centre Muraz	Abdoul-Salam Ouedraogo; Arsène Zongo; Essia Belarbi; Fabian Leendertz; Grit Schubert; Halidou Tinto; Lassana Sangaré; Soumeya Ouangraoua; Yacouba Sawadogo; Zekiba Tarnagda
EPI_ISL_1288215, EPI_ISL_1288383, EPI_ISL_1416681	Laboratorio Central de Epidemiología (LCE)	Instituto de Biotecnología de la UNAM	Alejandro Sanchez-Flores; Alfredo Herrera-Estrella; Alicia Ocaña-Mondragón; Angel Gustavo Salas-Lais; Bernardo Martínez-Miguel; Blanca Taboada; Brenda Irasema Maldonado-Meza; Carla Ivón Herrera-Najera; Carlos F. Arias; Celia Boukadida; Clara Esperanza Santacruz-Tinoco; Concepción Grajales-Muñiz; Consorcio Mexicano de Vigilancia Genómica (CoViGen-Mex). Authors (in alphabetical order): Julio Elias Alvarado-Yaah; Fernando Fontove-Herrera; Francisco Pulido; Gloria Elena Espinoza-Ayala; Gloria María Molina-Salinas; Gloria Vazquez; Hector Esteban Paz-Juárez; Hector Montoya-Fuentes; Helen Haydee Fernanda Ramírez-Plascencia; Jorge Ivan Salinal-Nevarez; José Antonio Enciso-Moreno; José Esteban Muñoz-Medina; José de Jesús Nuñez-Contreras; Juan Bautista Chale-Dzul; Luis Alberto Ochoa-Carrera; Margarita Matías-Florentino; María Guadalupe Santiago-Mauricio; María Guadalupe de Jesús Mireles-Rivera; Nelly Sélem-Mojica; Pavel Isa; Ricardo Grande; Santiago Ávila-Ríos; Víctor Hugo Borja-Aburto
EPI_ISL_1302341	Laboratorio Central de Epidemiología IMSS	Instituto de Biotecnología de la UNAM	Alejandra Hernández-Terán; Alejandro Sanchez-Flores; Alma Rincón-Rubio; Andrea Santos Coy-Arechavaleta; Authors from IBT; Blanca Taboada; Celia Boukadida; Clara Esperanza Santacruz-Tinoco; Edgar Mendieta-Condado; Eduardo Becerril-Vargas; Fidencio Mejía-Nepomuceno; Francisco Pulido; Gisela Barrera-Badillo; Gloria Vazquez; Hector Esteban Paz-Juárez; IMSS; InDRE and INER (in alphabetical order): Carlos F. Arias; Irma Lopez-Martinez; Jerome Jean Verleyen; Joel Armando Vázquez-Pérez; Jorge Salas-Hernández; José Arturo Martínez-Orozco; José Ernesto Ramírez-González; José Esteban Muñoz-Medina; Larissa Fernandes-Matano; Lucia Hernandez-Rivas; Luis Alberto Ochoa-Carrera; Margarita Matías-Florentino; Mario Mújica-Sánchez; Natividad Cruz-Ortiz; Pavel Isa; Ricardo Grande; Santiago Ávila-Ríos; Tatiana Nunez-Garcia; Teresita Rojas-Mendoza
EPI_ISL_1181607	Laboratorio Central de Saude Publica do Estado do Rio de Janeiro (LACEN-RJ)	Laboratory of Respiratory Viruses and Measles, Oswaldo Cruz Institute, FIOCRUZ	Alice Sampaio Rocha; Ana Carolina Mendonca; Andrea Cony Cavalcanti; Anna Carolina Paixao; Fernando Motta; Luciana Appolinario; Marilda Siqueira on behalf of the Fiocruz COVID-19 Genomic Surveillance Network; Paola Resende; Renata Serrano Lopes
EPI_ISL_1533989	Laboratorio Nacional de Salud	Laboratory of Respiratory Viruses and Measles, Oswaldo Cruz Institute, FIOCRUZ	Ana Carolina Mendonca; Anna Carolina Paixao; Cesar Roberto Conde Pereira; Claudia Estrada; Fernando Motta; Luciana Appolinario; Marilda Siqueira on behalf of the Fiocruz COVID-19 Genomic Surveillance Network; Paola Resende
EPI_ISL_833137	Laboratorio de Ecologia de Doencas Transmissiveis na Amazonia, Instituto Leonidas e Maria Deane - Fiocruz Amazonia	Laboratorio de Ecologia de Doencas Transmissiveis na Amazonia, Instituto Leonidas e Maria Deane - Fiocruz Amazonia	André Corado; Debora Duarte; Felipe Naveca on behalf of the Fiocruz COVID-19 Genomic Surveillance Network; Fernanda Nascimento; George Silva; Karina Pessoa; Luciana Gonçalves; Maria Júlia Brandão; Matilde Mejía; Michele Jesus; Valdinete Nascimento; Victor Souza; Ágatha Costa
EPI_ISL_1673323	Laboratorio de Investigaciones de Baney	Swiss Tropical and Public Health Institute	Bonifacio Manguire Nlavo; Carlos Cortes; Claudia Daubenberger; Diosdado Odjama Nseng Ada; Elizabeth Nyakarungu; Guillermo Garcia; Maximilian Mpina; Mitoha Ondo O Ayekaba; Philip Wonder Phiri; Philipp Wagner; Salome Hosch; Tobias Schindler
EPI_ISL_792350	Laboratorio del Hospital Interzonal General de Agudos Evita	Área de Secuenciación del Laboratorio de Virología del Hospital de Niños Dr. Ricardo Gutierrez on behalf of 'Proyecto Argentino Interinstitucional de genomica de SARS-CoV-2' (PAIS Consortium)	A; Desimone; E; Goya; Grossi; I; L; LE; Luczac; Lusso; MI; MS; Musto; Nabaes Jodar; Natale; O; S; Serrano; Valinotto; Viegas, M.
EPI_ISL_1396345	Laboratorio del Hospital Regional Ushuaia Gdor. Ernesto Campos	Nodo de Secuenciación Tierra del Fuego - Hospital Regional Ushuaia - Centro Austral De Investigaciones Científicas - Universidad Nacional De Tierra Del Fuego on behalf of 'Proyecto Argentino Interinstitucional de genomica de SARS-CoV-2' (PAIS Consortium)	Alejandro Ezequiel Rojas; Carina Andrea De Roccis; Carolina Beatriz Yulan; Cristina Fernanda Nardi; Fernando Gallego; Gabriel Alejandro Castro; Ivan Dario Gramundi; Manuel Fabian Boutureira; Santiago Guillermo Ceballos; Silvana Beatriz Cáceres
EPI_ISL_1020817, EPI_ISL_1038482, EPI_ISL_1161766	Laboratory Corporation of America	Respiratory Viruses Branch, Division of Viral Diseases, Centers for Disease Control and Prevention	Amanda Douglas; Amanda Suchanek; Andrea Throop; Ayla Burns; Ben L. Rambo-Martin; Bobbi Croy; Brian Krueger; Brian Norvell; Christos Petropoulos; Clinton R. Paden; Craig Lukasik; Dakota Howard; Dhwani Batra; Duncan MacCannell; Eyad Almasri Debbie Boles; Goran Stevovic; Howard Engler; Hrushikesh Deshmukh; Jake Humphrey; Jana Schroth; Joe Voshell; John Pruitt; Jonathan Meltzer; Jonathan Williams; Kimberly Wagner; Lax Iyer; Lyndon Tilson; Manoj Jain; Marcia Eisenberg; Mary Ann Cristobal; Mary Williamson; Michael Levandoski; Mike Sapeta; Mindy Nye; Minoo Agarwal; Mohan Kolli; Nuthawin Charoensri; Oren Cohen; Peter W. Cook; Prashant Gupta; Qian Zeng; Rama Ghatti; Scott Parker; Scott Ryan; Stanley Letovsky; Steven Ragan; Suresh Babu Selvaraju; Susan Countryman; Susan Hicks; Suxiang Tong; Suzanne Dale; Thomas Urban; Tim Kuphal; Tricia Zwiefelhofer; Vincent Drouillon
EPI_ISL_956326	Laboratory Medicine	Department of Laboratory Medicine, Lin-Kou Chang Gung Memorial Hospital, Taoyuan, Taiwan	Cheng-Hsun Chiu; Cheng-Ta Yang; Chung-Guei Huang; Guang-Wu Chen; Kuo-Chien Tsao; Kuo-Ming Lee; Mei-Jen Hsiao; Peng-Nien Huang; Po-Wei Huang; Shin-Ru Shih; Shu-Li Yang; Yi-Chun Liu; Yu-Nong Gong
EPI_ISL_1941490, EPI_ISL_1941508, EPI_ISL_1941512, EPI_ISL_1941520, EPI_ISL_1941522, EPI_ISL_1941528	Landesamt für Verbraucherschutz Sachsen Anhalt, Magdeburg	Institute of Medical Microbiology and Hospital Hygiene	Aljoscha Tersteegen; Prof. Dr. Achim Kaasch
EPI_ISL_1760006, EPI_ISL_1760007, EPI_ISL_1760008, EPI_ISL_1760009, EPI_ISL_1760020, EPI_ISL_1760021, EPI_ISL_1760022, EPI_ISL_1760023, EPI_ISL_2136815, EPI_ISL_2138821, EPI_ISL_2138822, EPI_ISL_2138832, EPI_ISL_2138848, EPI_ISL_2138852, EPI_ISL_2138854, EPI_ISL_2138857, EPI_ISL_2138861			
see above	Life Sciences Center, Vilnius University	Institute of Biotechnology, Life Sciences Center, Vilnius University	Albertas Timinskas; Alma Gedvilaite; Danguole Ziogiene; Emilija Vasiliunaite; Milda Norkiene
EPI_ISL_1510212	Life Sciences Center, Vilnius University	Vilnius University Hospital Santaros Klinikos, Center of Laboratory Medicine	Daniel Naumovas; Dovile Ezerskyte; Gytis Dudas; Ingrida Olendraite; Laimonas Griskevicius; Ligita Raugaite; Mindaugas Stoskus; Monika Katenaite; Rimvydas Norvilas
EPI_ISL_1718408, EPI_ISL_1985921, EPI_ISL_1986041, EPI_ISL_1986064, EPI_ISL_1986113, EPI_ISL_1986114, EPI_ISL_1986168, EPI_ISL_1986203			
see above	Lighthouse Lab in Alderley Park	Wellcome Sanger Institute for the COVID-19 Genomics UK (COG-UK) Consortium	Cordelia Langford; David K. Jackson; Dominic Kwiatkowski; Ewan Harrison; Ian Johnston; Jacquelyn Wynn; Jeffrey Barrett; John Sillitoe on behalf of the Wellcome Sanger Institute COVID-19 Surveillance Team; Mairead Hyland; Roberto Amato; Sonia Goncalves; The Lighthouse Lab in Alderley Park and Alex Alderton
EPI_ISL_1242199, EPI_ISL_1275749, EPI_ISL_1275972, EPI_ISL_1276088, EPI_ISL_1316127, EPI_ISL_1327538, EPI_ISL_1329214, EPI_ISL_1329219, EPI_ISL_1329268			
see above	Lighthouse Lab in Cambridge	Wellcome Sanger Institute for the COVID-19 Genomics UK (COG-UK) Consortium	Cordelia Langford; David K. Jackson; Dominic Kwiatkowski; Ewan Harrison; Ian Johnston; Jeffrey Barrett; John Sillitoe on behalf of the Wellcome Sanger Institute COVID-19 Surveillance Team; Rob Howes; Roberto Amato; Sonia Goncalves; The Lighthouse Lab in Cambridge and Alex Alderton
EPI_ISL_1939681, EPI_ISL_1986251, EPI_ISL_1986254, EPI_ISL_1986282, EPI_ISL_1986325, EPI_ISL_1986509, EPI_ISL_2238624			
see above	Lighthouse Lab in Glasgow	Wellcome Sanger Institute for the COVID-19 Genomics UK (COG-UK) Consortium	Anna Dominiczak and Alex Alderton; Carol Clugston; Cordelia Langford; David Gray; David K. Jackson; Dominic Kwiatkowski; Ewan Harrison; Harper VanSteenhouse; Ian Johnston; Jeffrey Barrett; John Sillitoe on behalf of the Wellcome Sanger Institute COVID-19 Surveillance Team; Roberto Amato; Sonia Goncalves; Yumi Kasai
EPI_ISL_1985756, EPI_ISL_2117910, EPI_ISL_2121107, EPI_ISL_2152803, EPI_ISL_2153043, EPI_ISL_2235679, EPI_ISL_2346864			
see above	Lighthouse Lab in Milton Keynes	Wellcome Sanger Institute for the COVID-19 Genomics UK (COG-UK) Consortium	Cordelia Langford; David K. Jackson; Dominic Kwiatkowski; Ewan Harrison; Ian Johnston; Jeffrey Barrett; John Sillitoe on behalf of the Wellcome Sanger Institute COVID-19 Surveillance Team; Roberto Amato; Sonia Goncalves; The Lighthouse Lab in Milton Keynes and Alex Alderton
EPI_ISL_601443	Lighthouse Lab in Milton Keynes	Wellcome Sanger Institute for the COVID-19 Genomics UK (COG-UK) consortium	Cordelia Langford; David K. Jackson; Dominic Kwiatkowski; Ewan Harrison; Ian Johnston; John Sillitoe on behalf of the Wellcome Sanger Institute COVID-19 Surveillance Team (http://www.sanger.ac.uk/covid-team); Roberto Amato; Sonia Goncalves; The Lighthouse Lab in Milton Keynes and Alex Alderton
EPI_ISL_2249639	M Health Fairview	Minnesota Department of Health, Public Health Laboratory	Alexandra Lorentz; Jacob Garfin; Matt Plumb; and Xiong Wang
EPI_ISL_1576771, EPI_ISL_1936184	MD PHL	MD PHL	Maryland Department of Health Laboratories Administration
EPI_ISL_1817033, EPI_ISL_2453875	MEPHI, Aix Marseille University	MEPHI, Aix Marseille University	Anthony LEVASSEUR
EPI_ISL_2482683, EPI_ISL_2482693, EPI_ISL_2482696, EPI_ISL_2676744, EPI_ISL_2676746, EPI_ISL_2676761, EPI_ISL_2676765			
see above	MIRIALIS CLUSES BECHET	CNR Virus des Infections Respiratoires - France SUD	Antonin Bal; Bruno Lina; Gregory Destras; Gwendolyne Burfin; Hadrien Regue; Laurence Josset; Martine Valette; Quentin Semanas

EPI_ISL_1469358, EPI_ISL_1469380	MRC/UVRI & LSHTM Uganda Research Unit	Where sequence data have been generated and submitted to GISAID	Dan Lule Bugembe; Isaac Sseeewanyana; Matthew Cotten; My V.T. Phan; Patrick Semanda; Pontiano Kaleebu; Susan Nabadda
EPI_ISL_1216076	MRCG at LSHTM Genomics lab	MRCG at LSHTM Genomics lab	Abdoulie Kanteh; Abdul Karim sesay; Bakary Sanyang; Jarra Manneh; Mariama Kujabi
EPI_ISL_1318472	MT Public Health Laboratory	Centers for Disease Control and Prevention Division of Viral Diseases, Pathogen Discovery	Anna Montmayeur; Anna Uehara; Ben L. Rambo-Martin; Clinton R. Paden; Dhwani Batra; Haibin Wang; Jasmine Padilla; Jing Zhang; Justin Lee; Katie Dillon; Krista Queen; Kristen Knipe; Kristine Lacek; Lori Rowe; Mark Burroughs; Matthew Schmerer; Mili Sheth; Peter W. Cook; Rachel Marine; Sam Shepard; Sarah Nobles; Shoshona Le; Suxiang Tong; Yan Li; Ying Tao
EPI_ISL_1482701	MUSC Molecular Pathology Laboratory	MUSC Molecular Pathology Laboratory	Dariusz Pytel; Frederick S. Nolte; Jaclyn Dunne; Julie W. Hirschhorn; Kristen Maurer; W. Bailey Glen Jr
EPI_ISL_2122527, EPI_ISL_2129571, EPI_ISL_2313265, EPI_ISL_2313306, EPI_ISL_2387453, EPI_ISL_2388214	MVZ Dr. Eberhard & Partner Dortmund	Robert Koch Institute	
EPI_ISL_2636756	MVZ Labor Dr. Fenner und Kollegen (Standort Hamburg)	Robert Koch Institute	
EPI_ISL_1847168, EPI_ISL_1847176, EPI_ISL_2259668, EPI_ISL_2259669, EPI_ISL_2390317	MVZ Labor Dr. Limbach & Kollegen GbR	Robert Koch Institute	
EPI_ISL_1936109, EPI_ISL_1936112, EPI_ISL_1936116, EPI_ISL_1936134	Main Chemical Laboratories Egypt Army	Main Chemical Laboratories Egypt Army	Abdullah Salama; AbedElrahman Zekri; Ahmed Gad; Mervat Hassan; Mohamed Seadawy; Mohamed Shamel; Sabah Ahmed
EPI_ISL_1922677, EPI_ISL_2319526	Maryland Genomics, Institute for Genome Sciences, University of Maryland School of Medicine	Maryland Genomics, Institute for Genome Sciences, University of Maryland School of Medicine	Aditya; Claire M; Fraser; Holly; Humphrys; Jacques; Kranthi; Lisa D; Luke J; Mehta; Mike; Ott; Ravel; Roussey; Sadzewicz; Sandra; Tallon; Vavikolanu
EPI_ISL_2094536	Max von Pettenkofer Institute, Virology, National Reference Center for Retroviruses, LMU Munich	Laboratory for Functional Genome Analysis; Dept. Genomics; Gene Center of the LMU Munich	Alexander Graf; Helmut Blum; Max Muenchhoff; Oliver Keppler; Stefan Krebs
EPI_ISL_2265478	Medizinische Laboratorien Düsseldorf	Robert Koch Institute	
EPI_ISL_2115390	Medizinisches Labor Wahl Ludenscheid	Robert Koch Institute	
EPI_ISL_2265010, EPI_ISL_2265019	Medizinisches Labor Wahl Lüdenscheid	Robert Koch Institute	
EPI_ISL_2448608, EPI_ISL_2448615	Microbiologia e Virologia	Istituto Zooprofilattico Sperimentale delle Venezie	Adelaide Milani; Alessia Schivo; Alice Fusaro; Ambra Pastori; Annalisa Salviato; Antonia Ricci; Calogero Terregino; Edoardo Giussani; Elisa Palumbo; Erika Giorgia Quaranta; Isabella Monne; Luca Tassoni
EPI_ISL_1583577, EPI_ISL_1583618, EPI_ISL_1761494, EPI_ISL_1761505	Microbiology Department, Laboratori Clínic Metropolitana Nord. Hospital Universitari Germans Trias i Pujol.	Can Ruti SARS-CoV-2 Sequencing Hub (HUGTiP/IrsiCaixa/IGTP)	Alba Sánchez; Anna Not; Antoni E Bordoy; Bonaventura Clotet; Cristina Casañ; Cristina Esteban; Francesc Catala-Moll; Gemma Clara; Ignacio Blanco; Marc Noguera-Julian; Maria Casadellà; Mariona Parera; Mercedes Guerrero; Montserrat Giménez; Pere-Joan Cardona; Pilar Armengol; Roger Paredes; Verónica Saludes; and Elisa Martró on behalf of the Can Ruti SARS-CoV-2 Sequencing Hub.
EPI_ISL_548145, EPI_ISL_1016855, EPI_ISL_1315311	Middlemore Hospital	Institute of Environmental Science and Research (ESR)	Anja Werno; Antje van der Linden; Arlo Upton; Chris Mansell; David Hammer; Dragana Drinkovic; Erasmus Smit; Gary McAuliffe; Hana Sofia Andersson; Hermes Perez; James Ussher; Jill Sherwood; Jing Wang; Joep de Ligt; Josh Freeman; Julia Howard; Juliet Elvy; Lauren Jelly; Mary DeAlmeida; Matt Blakiston; Matt Storey; Matthew Rogers; Max Bloomfield; Michael Addidle; Michelle Balm; Muhammad Faisal; Nikki Freed; Olin Silander; Olivia Stroeven; Rachel Boyle; Sally Roberts; SallyAnn Harbison; Sarah Jefferies; Sharmini Muttaiyah; Susan Morpeth; Susan Taylor; Timothy Blackmore; Vani Sathyendran; Veronica Playle; Virginia Hope; Xiaoyun Ren
EPI_ISL_718146	Ministry of Health Hospitals	Institute of Health and Community Medicine	Chan Chia Jui; Chua Hock Hin; David Perera; Ooi Mong How; Tonnii Sia Loong Loong; Wong Jyn Shan; Wong Kiing Aik
EPI_ISL_482959	Minnesota Department of Health, Public Health Laboratory	Minnesota Department of Health, Public Health Laboratory	Jacob Garfin; Matt Plumb; and Xiong Wang
EPI_ISL_1365031, EPI_ISL_1367678, EPI_ISL_1367685	Molecular diagnostic unit for viral haemorrhagic fevers and emerging viruses, Bouaké CHU Laboratory	Molecular diagnostic unit for viral haemorrhagic fevers and emerging viruses, Bouaké CHU Laboratory	Adjaratou Traoré; Bamba Fatoumata Touré; Chantal Akoua-Koffi; Coulibaly Mbegnan; Diané Bamourou; Essia Belarbi; Etilé Anoh; Fabian Leendertz; Grit Schubert; Kra Ouffoué; Monemo Pacome; Oby Wayoro; Safiatou Karidioula; Soundélé Maïté
EPI_ISL_1446920	NC State Laboratory of Public Health	Centers for Disease Control and Prevention Division of Viral Diseases, Pathogen Discovery	Alison Laufer Halpin; Ben L. Rambo-Martin; Clinton R. Paden; Dakota Howard; Darlene Wagner; Dave Wentworth; Dhwani Batra; Jasmine Padilla; Justin Lee; Katie Dillon; Krista Queen; Kristen Knipe; Kristine Lacek; Mark Burroughs; Matthew Schmerer; Mili Sheth; Peter Cook; Sam Shepard; Sarah Nobles; Shoshona Le; Suxiang Tong; Vivien Dugan; Yvette Unoarumhi
EPI_ISL_529742	NHLS-IALCH	KRISP, KZN Research Innovation and Sequencing Platform	Giandhari J; Khan S; Lessells R; Mdlalose K; Pillay S; Tegally H; Wilkinson E; York D; de Oliveira T
EPI_ISL_1540680, EPI_ISL_1540683	NMVRVI	Lithuanian University of Health Sciences Hospital, Department of Genetics and Molecular Medicine	Astra Vitkauskiene; Darius Cereskevicius; Inga Nasvytiene; Mantas Sarauskas; Marius Sukys; Rasa Ugenskiene; Renaldas Jurkevicius; Rima Vainoriene; Zivile Zemeckiene
EPI_ISL_1579527, EPI_ISL_1579571, EPI_ISL_1579607, EPI_ISL_1579784, EPI_ISL_1579903	NMVRVI	National Public Health Surveillance Laboratory	Ana Steponkiene; Danas Baksa; Jelena Razmuk; Lukas Vasionis; Lukas Zemaitis; Migle Gabrielaite; Svajune Muralyte
EPI_ISL_1579691, EPI_ISL_1656854, EPI_ISL_1656882, EPI_ISL_1911429	NVSPL	National Public Health Surveillance Laboratory	Ana Steponkiene; Arnoldas Pautienius; Astra Vitkauskiene; Danas Baksa; Dovydas Gecys; Ingrida Olendraite; Jelena Razmuk; Kamile Tamusauskaite; Laura Pareckaite; Lukas Vasionis; Lukas Zemaitis; Migle Gabrielaite; Svajune Muralyte; Vaiva Lesauskaite
EPI_ISL_1960165, EPI_ISL_1960181, EPI_ISL_1960198, EPI_ISL_1960606, EPI_ISL_1960607, EPI_ISL_1960608, EPI_ISL_1960609, EPI_ISL_1960610, EPI_ISL_1960611, EPI_ISL_1960612, EPI_ISL_1960613, EPI_ISL_1960614, EPI_ISL_1960615, EPI_ISL_1960616, EPI_ISL_2095985, EPI_ISL_2095990, EPI_ISL_2428377, EPI_ISL_2509615			
see above	Nacionaline visuomenes sveikatos prieziuros laboratorija	National Public Health Surveillance Laboratory	Ana Steponkiene; Danas Baksa; Jelena Razmuk; Lukas Vasionis; Lukas Zemaitis; Migle Gabrielaite; Svajune Muralyte
EPI_ISL_2334203, EPI_ISL_2334879, EPI_ISL_2428278, EPI_ISL_2694567, EPI_ISL_2694569, EPI_ISL_2694576	Nacionalinis maisto ir veterinarijos rizikos vertinimo institutas	National Public Health Surveillance Laboratory	Ana Steponkiene; Danas Baksa; Jelena Razmuk; Lukas Vasionis; Lukas Zemaitis; Migle Gabrielaite; Svajune Muralyte
EPI_ISL_1415344	National Centre For Cell Science	National Centre For Cell Science – INSACOG	Ajay Pillai; Dhiraj Paul; INSACOG Consortium team; Manoj Kumar Bhat; Mitali Inamdar; Mohak P Gujare; Shivang P. Bhanushali; Sonal Manik Chavan; Yogesh Shouche.
EPI_ISL_1909844, EPI_ISL_1909875	National Food and Veterinary Risk Assessment Institute (NMVRVI)	National Public Health Surveillance Laboratory	Ana Steponkiene; Danas Baksa; Jelena Razmuk; Lukas Vasionis; Lukas Zemaitis; Migle Gabrielaite; Svajune Muralyte
EPI_ISL_1407196	National HIV Reference Laboratory, Ministry of Health, Public Health Institute of Malawi	KRISP, KZN Research Innovation and Sequencing Platform	Auld A; Chilima B; Chiwaula M; Emmanuel SJ; Giandhari J; Kaba M; Kampira E; Kasambara W; Kim L; Lessells R; Maida A; Mvula B; Mwangomba W; Naidoo Y; Panja L; Pillay S; Tegally H; Wadonda N; Wilkinson E; de Oliveira T
EPI_ISL_862079	National Influenza Center, Virology Department	National Influenza Center	A Nejati; F Ajaminejad; J Yavarian; K Sadeghi; N Ghavvami and T Mokhtari Azad; NZ Shafiei Jandaghi; V Salimi
EPI_ISL_2285857	National Influenza Centre	National Influenza Centre	; Benjamiin B. Lindsey; Benjamin H. Foulkes; Dennis Laryea; Ernest Asiedu; Franklin Asiedu-Bekoe; Gordon Awandare; Ivy A. Asante; Joseph Oliver-Commey; Joyce Ngoi; Linda Boatemaa; Lorreta Kwasah; Mathew D. Parker; Michael Marks; Mildred Adusei-Poku; Sharon Hsu; Thushan I de Silva; William K. Ampofo
EPI_ISL_402125	National Institute for Communicable Disease Control and Prevention (ICDC) Chinese Center for Disease Control and Prevention (China CDC)	National Institute for Communicable Disease Control and Prevention (ICDC) Chinese Center for Disease Control and Prevention (China CDC)	Chen; Dai; F.-H.; Hu, Y.; J.-H.; J.-J.; J.-L. and Zhu; Liu, Y.; Pei; Q.-M.; She; Song; T.-Y.; Tao; Tian; Wang; Wang, W.; Wu, F.; Xu, L.; Y.-L.; Y.-M.; Y.-Y.; Y.-Z.; Yu, B.; Z.-G.; Z.-W.; Zhang; Zhao, S.; Zheng
EPI_ISL_1404880	National Institute for Food and Veterinary Risk Assessment	Lithuanian University of Health Sciences	Arnoldas Pautienius; Dovydas Gecys; Gediminas Alzbutas; Kamile Tamusauskaite; Lukas Zemaitis; Vaiva Lesauskaite
EPI_ISL_1910043, EPI_ISL_1910156, EPI_ISL_1910341, EPI_ISL_1911899	National Institute for Food and Veterinary Risk Assessment (NMVRVI)	National Public Health Surveillance Laboratory	Ana Steponkiene; Danas Baksa; Jelena Razmuk; Lukas Vasionis; Lukas Zemaitis; Migle Gabrielaite; Svajune Muralyte
EPI_ISL_469254	National Institute for Viral Disease Control and Prevention, China CDC	Institute of Viral Disease Control and Prevention, China CDC	Chun Huang; Dayan Wang; George Fu Gao; Guizhen Wu; Li Zhao; Lijuan Chen; Peihua Niu , Baoying Huang; Roujian Lu; Wenbo Xu; Wenjie Tan; Wenling Wang; Yubai Bi
EPI_ISL_498694, EPI_ISL_591277,	National Institute for Viral Disease Control and Prevention, China CDC	National Institute for Viral Disease Control and Prevention, China CDC	Cao Chen; Dayan Wang; George F.Gao; Hong Wang; Huilai Ma; Ji Wang; Jingdong Song; Jun Han; Kai Nie; LingLing Mao; Ruqin Gao; Shiwen Wang; Weimin Zhou; WenQing Yao , Wenbo Xu; Wenbo Xu; Wenjie Tan; Xiang Zhao; Yang Song; Yanhai Wang; Yao Meng; Yenan Feng; Yong Zhang; Yong Zhang , Bo Zhijian , Jianqun Zhang; Yuchao Wu; Zhaoguo Wang; Zhixiao Chen

EPI_ISL_850949, EPI_ISL_850951			
EPI_ISL_2324821	National Institute of Public Health	National Institute of Public Health	Alexander Nagy; Dusan Trnka; Helena Jirincova; Jaromira Vecerova; Timotej Suri
EPI_ISL_1261374	National Institute of Public Health	National Reference Laboratory for Influenza and Respiratory Viruses CZE	Alexander Nagy; Dusan Trnka; Helena Jirincova; Jaromira Vecerova; Timotej Suri
EPI_ISL_1828718, EPI_ISL_1971075	National Institute of Public Health	State Veterinary Institute Prague	A; D; H; J; Jirincova; Nagy; Suri; T; Trnka; Vecerova
EPI_ISL_1055886	National Laboratory for Health, Environment and Food	CISLD (Clinical Institute of Special Laboratory Diagnostics), University Children's Hospital, University Medical Center Ljubljana	Ana Grom; Barbara Jenko Bizjan; Jernej Kovač; Katarina Kozmos; Marko Pokorn; Maruša Debeljak; Robert Šket; Tadej Battelino; Tine Tesovnik
EPI_ISL_1112166	National Laboratory for Health, Environment and Food, OMM, Maribor	CISLD (Clinical Institute of Special Laboratory Diagnostics), University Children's Hospital, University Medical Center Ljubljana	Ana Grom; Barbara Jenko Bizjan; Jernej Kovač; Katarina Kozmos; Marko Pokorn; Maruša Debeljak; Robert Šket; Tadej Battelino; Tine Tesovnik
EPI_ISL_1195207	National Public Health Center, COVID Laboratory	National Public Health Center, National Biosafety Laboratory	Bernadett Pályi; Dániel Déri; Judit Henczkó; Norbert Solymosi; Nóra Magyar; Zoltán Kis
EPI_ISL_845545, EPI_ISL_845546, EPI_ISL_845548, EPI_ISL_845549, EPI_ISL_845550, EPI_ISL_845551, EPI_ISL_845552, EPI_ISL_845553, EPI_ISL_845554, EPI_ISL_845557, EPI_ISL_845558, EPI_ISL_845560, EPI_ISL_845561, EPI_ISL_845562, EPI_ISL_845563, EPI_ISL_845564, EPI_ISL_845565			
see above	National Public Health Laboratory, Cameroon	African Centre of Excellence for Genomics of Infectious Diseases (ACEGID), Redeemer's University	Oluniyi P.E. et al
EPI_ISL_1715187, EPI_ISL_1715188, EPI_ISL_1715190, EPI_ISL_1715191, EPI_ISL_1715192, EPI_ISL_1715193, EPI_ISL_1715194, EPI_ISL_1715195, EPI_ISL_1715196, EPI_ISL_1715197, EPI_ISL_1715199, EPI_ISL_1715201, EPI_ISL_1715203			
see above	National Public Health Laboratory, Cameroon	African Centre of Excellence for Genomics of Infectious Diseases, Redeemer's University	I.B. et al; Olawoye
EPI_ISL_479581, EPI_ISL_981024	National Public Health Laboratory, National Centre for Infectious Diseases	National Public Health Laboratory, National Centre for Infectious Diseases	Chavatte JM; Cui L; Lin Cui; Lin RTP; Mak TM; Octavia S; Raymond Tzer Pin Lin; Tze Minn Mak; Zhenyang Zhou; Zhou Z
EPI_ISL_2095980, EPI_ISL_2095981, EPI_ISL_2095982, EPI_ISL_2095983, EPI_ISL_2095984, EPI_ISL_2095986, EPI_ISL_2095987, EPI_ISL_2095988, EPI_ISL_2095989			
see above	National Public Health Surveillance Laboratory	National Public Health Surveillance Laboratory	Ana Steponkiene; Danas Baksa; Jelena Razmuk; Lukas Vasionis; Lukas Zemaitis; Migle Gabrielaite; Svajune Muralyte
EPI_ISL_1273393	National Reference Laboratory - Ministry of Health Maseru Lesotho	National Institute for Communicable Diseases of the National Health Laboratory Service	Amoako DG; Banda R; Bhiman JN; Gorova V; Ismail A; Mahlangu B; Mathabo M; Mohale T; Mooko M; Ntuli N; Scheepers C
EPI_ISL_1785368	National Virus Reference Laboratory	National Virus Reference Laboratory	Calum Walsh; Charlene Bennet; Cillian F De Gascun; Fiona Crispie; Gabriel Gonzalez; Jonathan Dean; Matthew McCabe; Michael Carr; Paul Cotter; Zoe Yandle
EPI_ISL_444999	Naval Health Research Center	Naval Medical Research Center Biological Defense Research Directorate	Adrian Paskey; Chris Myers; Dessiree Pena-Gomez; Ewell Hollis; Kimberly Bishop-Lilly; Kyle Long; Logan Voegtly; Melinda Balansay-Ames; Nathaniel Christy; Regina Cer; Roger Pan
EPI_ISL_1061033	New South Wales Health Pathology Royal Prince Alfred Hospital	Microbiology RPAH	Au, J.; Bull, R.; Deveson, I.; Foster, C.; Rawlinson, W.; Ruiz Silva, M.; Van Hal, S.
EPI_ISL_872606	Nigeria Centre for Disease Control (NCDC)	African Centre of Excellence for Genomics of Infectious Diseases (ACEGID), Redeemer's University	Oluniyi P.E. et al
EPI_ISL_527877	Nigeria Centre for Disease Control (NCDC)	African Centre of Excellence for Genomics of Infectious Diseases (ACEGID), Redeemer's University, Ede, Osun State, Nigeria	Oluniyi P.E. et al
EPI_ISL_579406	North Shore Hospital	Institute of Environmental Science and Research (ESR)	Anja Werno; Antje van der Linden; Arlo Upton; Chris Mansell; David Hammer; Dragana Drinkovic; Erasmus Smit; Gary McAuliffe; Hana Sofia Andersson; Hermes Perez; James Ussher; Jill Sherwood; Jing Wang; Joep de Ligt; Josh Freeman; Julia Howard; Juliet Elvy; Lauren Jelly; Mary DeAlmeida; Matt Blakiston; Matt Storey; Matthew Rogers; Max Bloomfield; Michael Addidle; Michelle Balm; Muhammad Faisal; Nikki Freed; Olin Silander; Sally Roberts; Sarah Jefferies; Sharmini Muttaiyah; Susan Morpeth; Susan Taylor; Timothy Blackmore; Vani Sathyendran; Veronica Playle; Virginia Hope; Xiaoyun Ren
EPI_ISL_1302680	Nucleic Acid Testing, National Reference Laboratory	GIGA Medical Genomics	Bouchra Boujemla; Esperence Umumararungu; Jacob Souopgui; Keith Durkin; Léon Mutesa; Maria Artesi; Marie-Pierre Hayette; Nathalie Renotte; Patrick Tuyisenge; Robert Rutayisire; Sabin Nsanzimana; Swaibu Gatare; Sébastien Bontems; Vincent Bours; Yvan Butera
EPI_ISL_1117426	Nucleo de Pesquisa em Inovacao Terapeutica - UFPE	LABBE, Federal University of Pernambuco	Bruno Sampaio; Heidi Lacerda Alves da Cruz; Maira Galdino da Rocha Pitta; Marco Katzenberger; Marcos da Silveira Regueira Neto; Michelly Cristiny Pereira; Reginaldo Goncalves de Lima Neto; Valdir de Queiroz Balbino; Wilson Jose da Silva Junior
EPI_ISL_1516300	OK Public Health Laboratory, Oklahoma State DOH	Centers for Disease Control and Prevention Division of Viral Diseases, Pathogen Discovery	Alison Laufer Halpin; Ben L. Rambo-Martin; Clinton R. Paden; Dakota Howard; Darlene Wagner; Dave Wentworth; Dhwani Batra; Jasmine Padilla; Justin Lee; Katie Dillon; Krista Queen; Kristen Knipe; Kristine Lacek; Mark Burroughs; Matthew Schmerer; Mili Sheth; Peter Cook; Sam Shepard; Sarah Nobles; Shoshona Le; Suxiang Tong; Vivien Dugan; Yvette Unoarumhi
EPI_ISL_2545564	OKMI FN Brno	CEITEC MU	Bystry V.; Deissova T.; Dolejska M.; Machackova T.; Pardy F.; Zdrazilova Dubska L.
EPI_ISL_1620228	OLVZ Aalst	OLVZ Aalst	Astrid Holderbeke
EPI_ISL_1366745	OUCRU	OUCRU	Guy Thwaites; Huynh Trung Trieu; Lam Minh Yen; Le Manh Hung; Le Nguyen Truc Nhu; Le Thi Thu Huong; Le Van Tan; Nghiem My Ngoc; Ngo Ngoc Quang Minh; Nguyen Thanh Dung; Nguyen Thanh Phong; Nguyen Thanh Truong; Nguyen Thi Thu Hong; Nguyen To Anh; Nguyen Tri Dung; Nguyen Van Vinh Chau; Tran Nguyen Hoang Tu; Tran Tan Thanh
EPI_ISL_492020	Oman-NIC	Department of Microbiology and Immunology-SQUH	Abdulla Balkhair; Ahlam Al-Amri; Aisha Al-Amri; Aisha Al-Busaidi; Amina Al Jardani; Fahad Zadjali; Fatma BaAlawi; Hamida AL Barwani; Hanan Al-kindi; Intisar Al-Shukri; Khulood Al-Mammary; Mohammed Al-Tobi; Samiha Al Kharusi; Samira Al-Maruqi; Zeyana AL-Dahmani
EPI_ISL_1979313, EPI_ISL_1979568, EPI_ISL_1979626, EPI_ISL_1979648	Originating lab: Wales Specialist Virology Centre Sequencing lab: Pathogen Genomics Unit	Public Health Wales Microbiology Cardiff Wales Specialist Virology Centre	Alec Birchley; Alexander Adams; Amy Gaskin; Angela Marchbank; Bree Gatica-Wilcox; Catherine Moore; Jason Coombes; Joanne Watkins; Joel Southgate; Johnathan Evans; Laura Gifford; Lauren Gilbert; Lee Graham; Malorie Perry; Matthew Bull; Nicole Pacchiarini; Sally Corden; Sara Kumziene-Summerhayes; Sara Rey; Sarah Taylor; Simon Cottrell; Sophie Jones; Tom Connor
EPI_ISL_1490266	Ostfold Hospital Trust - Kalnes, Centre for Laboratory Medicine, Section for gene technology and infection serology	Norwegian Institute of Public Health, Department of Virology	Atiya R Ali; Debech Nadia; Engebretsen Serina Beate; Garcia Llorente Ignacio; Hilde Elshaug; Hilde Vollan; Jon Bråte; Kamilla Heddeland Instefjord; Karoline Bragstad; Kathrine Stene-Johansen; Marie Paulsen Madsen; Olav Hungnes; Pedersen Benedikte Nevjen; Rasmus Riis Kopperud
EPI_ISL_1543465, EPI_ISL_1543808	Pandemic Response Lab - NYC	Pandemic Response Lab, R&D	Cybill del Castillo; Dylan Law; Haiping Hao; Henry Lee; Jon Laurent; Katharine Nelson; Melissa Hopkins; Michael Hammerling; Pradeep Bugga; Shinyoung Clair Kang; Sol Rey; William Ward
EPI_ISL_810801, EPI_ISL_810804	PathWest Laboratory Medicine WA	PathWest Laboratory Medicine WA Microbial Surveillance Unit	PathWest Laboratory Medicine WA Microbial Surveillance Unit
EPI_ISL_712081	Port Elizabeth Provincial Hospital, National Health Laboratory Services, Eastern Cape, South Africa	National Institute for Communicable Diseases of the National Health Laboratory Service	Allam M; Bhiman JN; Ismail A; Mahlangu B; Mohale T; Ntuli N
EPI_ISL_2171204, EPI_ISL_2188829	Prime Care Alpha Covid-19 Testing Laboratory	Philippine Genome Center	Alethea R. de Guzman; Anna Ong-Lim; Arianne A. Zamora; Asia Louisa U. Chong; Benedict A. Maralit; Candice Francheska B. Tambaoan; Carlo M. Lapid; Celia Carlos; Devon Ray Pacial; Edsel Maurice Salvaña; El King D. Morado; Elcid Aaron R. Pangilinan; Eva Maria Cutiongco-de la Paz; Francis A. Tablizo; Irish Coleen A. Asin; Jaime C. Montoya; Jan Michael C. Yap; Jo-Hannah S. Llames; John Q. Wong; Joshua Gregor A. Dizon; Juan Antonio R. Magalang; Karol Sophia Agape R. Padilla; Kenneth M. Kim; Kris P. Punayan; Marc Edsel C. Ayes; Marc Jerrone R. Castro; Maria Rosario Singh-Vergeire and Cynthia P. Saloma; Maria Sofia L. Yangzon; Marissa Alejandria; Razel Nikka M. Hao; Renato Jacinto Q. Mantaring; Rianna Patricia S. Cruz; Sheila Mae M. Araiza
EPI_ISL_849747	Public Health Virology Laboratory, Forensic and Scientific Services (PHV-FSS)	Public Health Virology Laboratory, Forensic and Scientific Services (PHV-FSS)	Son Nguyen et al
EPI_ISL_1300526	Public Health Virology-Forensic and Scientific Services (PHV-FSS)	Public Health Virology-Forensic and Scientific Services (PHV-FSS)	Son Nguyen
EPI_ISL_950607	Queens Medical Centre, Clinical Microbiology Department / DeepSeq Nottingham	COVID-19 Genomics UK (COG-UK) Consortium	Christopher Moore; Fei Sang; Gemma Clark; Hannah Howson-Wells; Johnny Debebe; Jonathan Ball; Joseph Chappell; Manjinder Khakh; Matthew Carlisle; Matthew Loose; Michelle M Lister; Nadine Holmes; Patrick McClure; Theocharis Tsoleridis; Vicki M Fleming; Victoria Wright; Wendy Smith
EPI_ISL_530250	Queensland Health Forensic and Scientific Services, Public Health Virology	Public Health Virology Laboratory, Forensic and Scientific Services, Queensland Health	Son Nguyen et al
EPI_ISL_1424529, EPI_ISL_1424599	Queensland Medical Laboratories	Victorian Infectious Diseases Reference Laboratory (VIDRL) and the Melbourne Diagnostic Unit Public Health Laboratory (MDU-PHL)	N.L.; Palou, T.; Seemann, T.; Sherry; Vaccher, S.
EPI_ISL_572163	Quest Diagnostics	Quest Diagnostics	Anderson, B.; D.F.; Gerasimova, A.; Grover, D.; Hua, M.; K.E.; Kagan; Lacbawan, F.; Liu Y.; Livingston; Owen, R.; R.M.; Rosenthal; S.H.; Shalhout
EPI_ISL_1253511, EPI_ISL_1314567	Quest Diagnostics Incorporated	Centers for Disease Control and Prevention Division of Viral Diseases, Pathogen Discovery	A. Gerasimova; A. Perez; B. Anderson; Ben L. Rambo-Martin; Clinton R. Paden; Dakota Howard; Dhwani Batra; Duncan MacCannell; F. Lacbawan; I. A. Shlyakhter; K.E. Livingston; L.E. Bernstein; M. Hua; P. Tanpaiboon; Peter W. Cook; R. M. Kagan; R. Owen; R. V. Rolando; S. H. Rosenthal; Suxiang Tong; Y. Liu
EPI_ISL_1986751	Randox Laboratories	Wellcome Sanger Institute for the COVID-19 Genomics UK (COG-UK) Consortium	Cordelia Langford; David K. Jackson; Dominic Kwiatkowski; Ewan Harrison; Ian Johnston; Jeffrey Barrett; John Sillitoe on behalf of the Wellcome Sanger Institute COVID-19 Surveillance Team; Randox Laboratories and Alex Alderton; Roberto Amato; Sonia Goncalves
EPI_ISL_2095895	RezusLT	National Public Health Surveillance Laboratory	Ana Steponkiene; Danas Baksa; Jelena Razmuk; Lukas Vasionis; Lukas Zemaitis; Migle Gabrielaite; Svajune Muralyte
EPI_ISL_779409	Royal Darwin Hospital Pathology	MDU-PHL	Caly L.; Druce J.; M.L.; Meumann, E.; N.L.; Sait; Seemann T.; Sherry
EPI_ISL_2289616	SELARL MIRIALIS	CNR Virus des Infections Respiratoires - France SUD	Antonin Bal; Bruno Lina; Gregory Destras; Gwendolyne Burfin; Hadrien Regue; Laurence Josset; Martine Valette; Quentin Semanas
EPI_ISL_1623167,	SELARL MIRIALIS CLUSES	CNR Virus des Infections Respiratoires - France SUD	Antonin Bal; Bruno Lina; Gregory Destras; Gwendolyne Burfin; Hadrien Regue; Laurence Josset; Martine Valette; Quentin Semanas

EPI_ISL_1623586			
EPI_ISL_1241728	SYNLAB	GIGA Medical Genomics	Bouchra Boujemla; Cécile Meex; Keith Durkin; Maria Artesi; Marie-Pierre Hayette; Nathalie Renotte; Pierrette Melin; Raphaël Boreux; Sébastien Bontems; Vincent Bours
EPI_ISL_1823201, EPI_ISL_2428545	SYNLAB Lietuva UAB	Vilnius University Hospital Santaros Klinikos, Center of Laboratory Medicine	Daniel Naumovas; Dovile Ezerskyte; Gytis Dudas; Ingrida Olendraite; Laimonas Griskevicius; Ligita Raugaite; Mindaugas Stoskus; Monika Katenaite; Rimvydas Norvilas
EPI_ISL_2469565	SYNLAB MVZ Leinfelden-Echterdingen	Robert Koch Institute	
EPI_ISL_1214521	SYNLAB MVZ Leverkusen	Robert Koch Institute	
EPI_ISL_1284252, EPI_ISL_2118288, EPI_ISL_2124462	SYNLAB MVZ Trier	Robert Koch Institute	
EPI_ISL_1845707, EPI_ISL_1852817	SYNLAB MVZ Weiden	Robert Koch Institute	
EPI_ISL_749906	Sanatorio Americano	Institut Pasteur de Montevideo	Ana Carolina Mendonça; Andrés Lizasoain; Camila Simoes; Cecilia Alonso; Cecilia Salazar; Daiana Mir; Fernando López-Tort; Fernando Motta; Gonzalo Bello; Ighor Arantes; Ignacio Ferrés; Jose Sotelo; Leticia Maya; Letícia Garay Martins; Luciana Appolinario; Lucía Spangenberg; Mailen Arleo; Mariana Brandes; Marilda Mendonça Siqueira; Marilda Tereza Mar da Rosa; María José Benitez-Galeano; Martín Graña; Matías Castells; Matías Victoria; Matías Salvo; Natalia Rego; Natalia Reyes; Pablo Smircich; Paola Cristina Resende; Rodney Colina; Tamara Fernandez-Calero; Tania Possi; Tatiana Schäffer Gregianini; Verónica Noya; Yasser Vega
EPI_ISL_1468846	Sharp HealthCare Laboratory	Andersen lab at Scripps Research	Art Mendoza; Cathy Woerle; Jacquelyn Berumen; Liam McGinnis; Omid Bakhtar; SEARCH Alliance San Diego with Aaron Harding
EPI_ISL_1533610	Shiraz University	Shiraz University	Abozar Ghorbani; Ebrahim Eftekhar; Hakimeh Ahmadi; Hamed Gouklani
EPI_ISL_583896	Singapore General Hospital	Department of Microbiology	Chenhao Li; Karrie Ko; Kenneth Xin Long Chan; Kern Rei Chng; Kian Sing Chan; Kun Lee Lim; Lynette Oon; Niranjan Nagarajan; Nurdyana Abdul Rahman; Sui Sin Goh
EPI_ISL_1844130, EPI_ISL_1844259	Sonic - MVZ Medizinisches Labor Bremen GmbH	Robert Koch Institute	
EPI_ISL_1363477	Sonora Quest Laboratories	TGen North	Ashlyn Pfeiffer; Chris French; Darrin Lemmer; Dave Engelthaler; Hayley Yaglom; Heather Centner; Jolene Bowers; The Arizona COVID Genomics Union (ACGU)
EPI_ISL_455077	South Eastern Area Laboratory Services	NSW Health Pathology - Institute of Clinical Pathology and Medical Research; Westmead Hospital; University of Sydney	CIDM-PH et al.
EPI_ISL_740868	South Eastern Area Laboratory Services (SEALS)	NSW Health Pathology - Institute of Clinical Pathology and Medical Research; Westmead Hospital; University of Sydney	CIDM-PH et al.
EPI_ISL_1292663	State Laboratories Division, Hawaii State Department of Health	State Laboratories Division, Hawaii State Department of Health	Ayana Garnet; Drew Kuwazaki; Edward Desmond; Pamela O'Brien; Razvan Sultana
EPI_ISL_1417511, EPI_ISL_2210205	Swedish national genomic surveillance program of SARS-CoV-2	The Public Health Agency of Sweden	Alma Brolund; Maria Lind Karlberg; Maximilian Riess; Swedish national genomic surveillance program of SARS-CoV-2
EPI_ISL_1988255, EPI_ISL_1988263, EPI_ISL_1988286, EPI_ISL_1988288, EPI_ISL_1988300, EPI_ISL_1988301, EPI_ISL_1988308, EPI_ISL_1988328, EPI_ISL_1988341, EPI_ISL_1988395, EPI_ISL_1988437, EPI_ISL_1988462, EPI_ISL_1988503, EPI_ISL_1988508, EPI_ISL_1988519, EPI_ISL_1988562, EPI_ISL_1988611, EPI_ISL_1988621, EPI_ISL_1988631, EPI_ISL_1988640			
see above	Synlab Haut de France	UMR 8199/1283 EGID	Derhourhi Mehdi
EPI_ISL_1566470, EPI_ISL_1566566, EPI_ISL_1568272	Synlab MVZ Augsburg	Robert Koch Institute	
EPI_ISL_1591299	Teaching Institute for Public Health of Split-Dalmatia County	Croatian Institute of Public Health	Irena Tabain; Ivana Ferenčak
EPI_ISL_2694568	UAB "Baltic Medics"	National Public Health Surveillance Laboratory	Ana Steponkiene; Danas Baksa; Jelena Razmuk; Lukas Vasionis; Lukas Zemaitis; Migle Gabrielaite; Svajune Muralyte
EPI_ISL_2497502, EPI_ISL_2497505, EPI_ISL_2497531, EPI_ISL_2497538, EPI_ISL_2625912, EPI_ISL_2625924, EPI_ISL_2644773			
see above	UAB "Rezus.lt"	Institute of Biotechnology, Life Sciences Center, Vilnius University	Albertas Timinskas; Alma Gedvilaite; Danguole Ziogiene; Emilija Vasiliunaite; Emilija Vasiliunaite__ Milda Norkiene__ Danguole Ziogiene__ Albertas Timinskas__ Alma Gedvilaite; Milda Norkiene
EPI_ISL_2334905, EPI_ISL_2334906, EPI_ISL_2509532, EPI_ISL_2693984, EPI_ISL_2694022, EPI_ISL_2694542, EPI_ISL_2694563, EPI_ISL_2694564, EPI_ISL_2694565, EPI_ISL_2694575			
see above	UAB "Rezus.lt"	National Public Health Surveillance Laboratory	Ana Steponkiene; Danas Baksa; Jelena Razmuk; Lukas Vasionis; Lukas Zemaitis; Migle Gabrielaite; Svajune Muralyte
EPI_ISL_2428700, EPI_ISL_2428929	UAB "Rezus.lt"	Vilnius University Hospital Santaros Klinikos, Center of Laboratory Medicine	Daniel Naumovas; Dovile Ezerskyte; Gytis Dudas; Ingrida Olendraite; Ligita Raugaite; Monika Katenaite; Rimvydas Norvilas
EPI_ISL_1756769	UAB Anteja Diagnostics Laboratory	Vilnius University Hospital Santaros Klinikos, Center of Laboratory Medicine	Daniel Naumovas; Dovile Ezerskyte; Gytis Dudas; Ingrida Olendraite; Laimonas Griskevicius; Ligita Raugaite; Mindaugas Stoskus; Monika Katenaite; Rimvydas Norvilas
EPI_ISL_2625891, EPI_ISL_2625892, EPI_ISL_2644782, EPI_ISL_2644798	UAB Diagnostikos laboratorija	Institute of Biotechnology, Life Sciences Center, Vilnius University	Albertas Timinskas; Alma Gedvilaite; Danguole Ziogiene; Emilija Vasiliunaite; Emilija Vasiliunaite__ Milda Norkiene__ Danguole Ziogiene__ Albertas Timinskas__ Alma Gedvilaite; Milda Norkiene
EPI_ISL_2334187, EPI_ISL_2509571, EPI_ISL_2614788	UAB Diagnostikos laboratorija	National Public Health Surveillance Laboratory	Ana Steponkiene; Danas Baksa; Jelena Razmuk; Lukas Vasionis; Lukas Zemaitis; Migle Gabrielaite; Svajune Muralyte
EPI_ISL_2428683	UAB Diagnostikos laboratorija	Vilnius University Hospital Santaros Klinikos, Center of Laboratory Medicine	Daniel Naumovas; Dovile Ezerskyte; Gytis Dudas; Ingrida Olendraite; Ligita Raugaite; Monika Katenaite; Rimvydas Norvilas
EPI_ISL_2334588, EPI_ISL_2334661, EPI_ISL_2428387, EPI_ISL_2509802, EPI_ISL_2694106, EPI_ISL_2694578	UAB InMedica	National Public Health Surveillance Laboratory	Ana Steponkiene; Danas Baksa; Jelena Razmuk; Lukas Vasionis; Lukas Zemaitis; Migle Gabrielaite; Svajune Muralyte
EPI_ISL_1756904, EPI_ISL_1823197, EPI_ISL_1823198, EPI_ISL_1823199, EPI_ISL_1969180, EPI_ISL_2428633	UAB InMedica	Vilnius University Hospital Santaros Klinikos, Center of Laboratory Medicine	Daniel Naumovas; Dovile Ezerskyte; Gytis Dudas; Ingrida Olendraite; Laimonas Griskevicius; Ligita Raugaite; Mindaugas Stoskus; Monika Katenaite; Rimvydas Norvilas
EPI_ISL_2625896, EPI_ISL_2625898, EPI_ISL_2625903, EPI_ISL_2625906, EPI_ISL_2625909, EPI_ISL_2625911, EPI_ISL_2625922, EPI_ISL_2644783, EPI_ISL_2644793, EPI_ISL_2644818			
see above	UAB Medicina practica laboratorija	Institute of Biotechnology, Life Sciences Center, Vilnius University	Albertas Timinskas; Alma Gedvilaite; Danguole Ziogiene; Emilija Vasiliunaite; Emilija Vasiliunaite__ Milda Norkiene__ Danguole Ziogiene__ Albertas Timinskas__ Alma Gedvilaite; Milda Norkiene
EPI_ISL_1576950, EPI_ISL_2006715, EPI_ISL_2227185, EPI_ISL_2428501, EPI_ISL_2497851, EPI_ISL_2497856, EPI_ISL_2497860, EPI_ISL_2497863			
see above	UAB Medicina practica laboratorija	Lithuanian University of Health Sciences	Arnoldas Pautienius; Daniel Naumovas; Dovile Ezerskyte; Dovydas Gecys; Gediminas Alzbutas; Gytis Dudas; Ingrida Olendraite; Kamile Tamusauskaite; Laimonas Griskevicius; Ligita Raugaite; Lukas Zemaitis; Mindaugas Stoskus; Monika Katenaite; Rimvydas Norvilas; Vaiva Lesauskaite
EPI_ISL_2509666, EPI_ISL_2509872, EPI_ISL_2694566, EPI_ISL_2694570, EPI_ISL_2694571, EPI_ISL_2694572, EPI_ISL_2694577			
see above	UAB Medicina practica laboratorija	National Public Health Surveillance Laboratory	Ana Steponkiene; Danas Baksa; Jelena Razmuk; Lukas Vasionis; Lukas Zemaitis; Migle Gabrielaite; Svajune Muralyte
EPI_ISL_2448513	ULSS 1 Dolomiti	Istituto Zooprofilattico Sperimentale delle Venezie	Adelaide Milani; Alessia Schivo; Alice Fusaro; Ambra Pastori; Annalisa Salviato; Antonia Ricci; Calogero Terregino; Edoardo Giussani; Elisa Palumbo; Erika Giorgia Quaranta; Isabella Monne; Luca Tassoni
EPI_ISL_1490697	Unity Health Toronto	Ontario Institute for Cancer Research	Bernard Lam; Felicia Vincelli; Ilinca Lungu; Jared T. Simpson; Jeremy Johns; Karel Boissinot; Larissa M. Matukas; Le Luu; Mark Downing; Paul Krzyzanowski; Philip Zuzarte; Ramzi Fattouh; Richard de Borja; Samira Mubareka; TIBDN; Trina Otterman; Yan Chen
EPI_ISL_1975115	University College London, Great Ormond Street Hospital for Children NHS Foundation Trust, Imperial College Healthcare NHS Trust	COVID-19 Genomics UK (COG-UK) Consortium	Alison Holmes; Charlotte Williams; Helena Tutill; Jacqueline Findlay; James Price; Judith Breuer; Julianne Brown; Kathryn Harris; Leysa Forrest; Mark Kristiansen; Paola Niola; Paola Resende Silva; Patricia Dyal; Paul Randell; Rachel Williams; Samuel Weeks; Sergi Castellano; Sunando Roy; Tony Brooks; Yasmin Panchbhaya
EPI_ISL_710570	University Hospital Dubrava	Ruđer Bošković Institute; Forensic Science Centre Ivan Vučetić; University of Zagreb Faculty of Science	Ana Livun; Antonela Blažeković; Boris Maček; Danilo Licastro; Dunja Glavaš; Fran Borovečki; Fuad Ćosović; Gordana Maravić Vlahoviček; Ivan Šamija; Ivana Ćelap; Jasna Kašman; Josipa Skelin; Katarina Marija Tupek; Kristian Vlahoviček; Kristina Gotovac Jereić; Lidija Cvetko-Krajinović; Lucija Basić; Lucija Markulin; Maja Kuzman; Marina Korolija; Mario Štefanović; Mirjana Domazet-Lošo; Paula Štancl; Petra Vrabec; Robert Beližić; Rosa Karlić; Sanja Tadinac; Senčica Pejša; Tomislav Domazet-Lošo; Valentina Đumlijan-Combaj; Vjekoslav Tomaić; Vladimir Krajinović; Željka Maček Šafranko
EPI_ISL_1369646, EPI_ISL_2104500, EPI_ISL_2104501, EPI_ISL_2104631, EPI_ISL_2343073, EPI_ISL_2343182, EPI_ISL_2343183, EPI_ISL_2601073			
see above	University Hospitals of Geneva, Laboratory of Virology	HUG, Laboratory of Virology and the Health2030 Genome Center	Ana Rita Goncalves; Deborah Penet; Emmanouil Dermitzakis; Henri Pegeot; Ioannis Xenarios; Keith Harshman; Laurent Kaiser; Lorenzo Cerutti; Melyssa Elies; Samuel Cordey
EPI_ISL_2365937	University of Bari Biomedical Sciences and Human Oncology-Policlinico	University of Bari Biomedical Sciences and Human Oncology	Accogli M.; Chironna M.; Loconsole D.; sallustio A.
EPI_ISL_671437	University of Debrecen, Department of Medical Microbiology	National Laboratory of Virology, Szentágothai Research Centre	Balázs Somogyi; Brigitta Zana; Endre Gábor Tóth; Eszter Csoma; Ferenc Jakab; Gábor Kemenesi

EPI_ISL_2612806	University of Liège COVID-19 testing center	GIGA Medical Genomics	Bouchra Boujemla; Cécile Meex; Fabrice Bureau; Keith Durkin; Laurent Gillet; Maria Artesi; Marie-Pierre Hayette; Nathalie Renotte; Sébastien Bontems; Vincent Bours; Wouter Coppieters
EPI_ISL_590728	University of Michigan Clinical Microbiology Laboratory	Lauring Lab, University of Michigan, Department of Microbiology and Immunology	Valesano
EPI_ISL_977352	University of Zambia, School of Veterinary Medicine	UNZAVET and PATH	Daniel Bridges; Mulenga Mwenda-Chimfwembe; Ngonda Saasa
EPI_ISL_708815	Urban Institute for Disease Prevention and Control	National Institute of Health, Department of Medical Sciences, Ministry of Public Health, Thailand	Malinee Chittaganpitch; Pakorn Piromtong; Pilailuk Okada; Siripaporn Phuygun; Sittiporn Parnmen; Sunthareeya Waicharoen; Thanutsapa Thanadachakul; Warawan Wongboot
EPI_ISL_1960617	Viesoji istaiga Klaipedos universitetine ligonine	National Public Health Surveillance Laboratory	Ana Steponkiene; Danas Baksa; Jelena Razmuk; Lukas Vasionis; Lukas Zemaitis; Migle Gabrielaite; Svajune Muralyte
EPI_ISL_2428461, EPI_ISL_2428474, EPI_ISL_2428485, EPI_ISL_2644812, EPI_ISL_2644813, EPI_ISL_2644819	Viesoji istaiga Respublikine Siauliu ligonine	Institute of Biotechnology, Life Sciences Center, Vilnius University	Albertas Timinskas; Alma Gedvilaite; Danguole Ziogiene; Emilija Vasiliunaite; Milda Norkiene
EPI_ISL_2615051, EPI_ISL_2694039, EPI_ISL_2694040, EPI_ISL_2694071, EPI_ISL_2694533, EPI_ISL_2694573	Viesoji istaiga Respublikine Siauliu ligonine	National Public Health Surveillance Laboratory	Ana Steponkiene; Danas Baksa; Jelena Razmuk; Lukas Vasionis; Lukas Zemaitis; Migle Gabrielaite; Svajune Muralyte
EPI_ISL_2625886, EPI_ISL_2625894	Viesoji istaiga Vilniaus universiteto ligonine Santaros klinikos	Institute of Biotechnology, Life Sciences Center, Vilnius University	Emilija Vasiliunaite__ Milda Norkiene__ Danguole Ziogiene__ Albertas Timinskas__ Alma Gedvilaite
EPI_ISL_2428226, EPI_ISL_2428316, EPI_ISL_2428383, EPI_ISL_2615175	Viesoji istaiga Vilniaus universiteto ligonine Santaros klinikos	National Public Health Surveillance Laboratory	Ana Steponkiene; Danas Baksa; Jelena Razmuk; Lukas Vasionis; Lukas Zemaitis; Migle Gabrielaite; Svajune Muralyte
EPI_ISL_2510701	Viesoji istaiga Vilniaus universiteto ligonine Santaros klinikos	Vilnius University Hospital Santaros Klinikos, Center of Laboratory Medicine	Daniel Naumovas; Dovile Ezerskyte; Gytis Dudas; Ingrida Olendraite; Ligita Raugaite; Monika Katenaite; Rimvydas Norvilas
EPI_ISL_2226596, EPI_ISL_2226603, EPI_ISL_2226604, EPI_ISL_2226632	Vilniaus universitetas	Institute of Biotechnology, Life Sciences Center, Vilnius University	Albertas Timinskas; Alma Gedvilaite; Danguole Ziogiene; Emilija Vasiliunaite; Milda Norkiene
EPI_ISL_2095896, EPI_ISL_2095897, EPI_ISL_2334977	Vilniaus universitetas	National Public Health Surveillance Laboratory	Ana Steponkiene; Danas Baksa; Jelena Razmuk; Lukas Vasionis; Lukas Zemaitis; Migle Gabrielaite; Svajune Muralyte
EPI_ISL_1585832, EPI_ISL_1585833, EPI_ISL_1661662, EPI_ISL_1661747, EPI_ISL_1823196, EPI_ISL_1823200	Vilnius University Hospital Santaros Klinikos	Vilnius University Hospital Santaros Klinikos, Center of Laboratory Medicine	Daniel Naumovas; Dovile Ezerskyte; Gytis Dudas; Ingrida Olendraite; Laimonas Griskevicius; Ligita Raugaite; Mindaugas Stoskus; Monika Katenaite; Rimvydas Norvilas
EPI_ISL_1661639, EPI_ISL_1661694, EPI_ISL_1661705, EPI_ISL_1661716, EPI_ISL_1661727, EPI_ISL_1661738	Vilnius University Hospital Santaros Klinikos, Center of Laboratory Medicine	Vilnius University Hospital Santaros Klinikos, Center of Laboratory Medicine	Daniel Naumovas; Dovile Ezerskyte; Gytis Dudas; Ingrida Olendraite; Laimonas Griskevicius; Ligita Raugaite; Mindaugas Stoskus; Monika Katenaite; Rimvydas Norvilas
EPI_ISL_802863	Vilnius University Hospital Santaros Klinikos, Vilnius University	Institute of Biotechnology, Life Sciences Center, Vilnius University	Albertas Timinskas; Alma Gedvilaite; Aurelija Zvirbliene; Daniel Naumovas; Emilija Vasiliunaite; Laimonas Griskevicius; Milda Norkiene
EPI_ISL_1908649, EPI_ISL_2339513	Vilnius University, Life Sciences Center	Vilnius University Hospital Santaros Klinikos, Center of Laboratory Medicine	Daniel Naumovas; Dovile Ezerskyte; Gytis Dudas; Ingrida Olendraite; Laimonas Griskevicius; Ligita Raugaite; Mindaugas Stoskus; Monika Katenaite; Rimvydas Norvilas
EPI_ISL_1914218, EPI_ISL_1914219	Viollier AG	Department of Biosystems Science and Engineering, ETH Zurich	Andrea Patrignani; Andreia Cabral de Gouvea; Catharine Aquino; Chaoran Chen; Christiane Beckmann; Christoph Noppen; David Dreifuss; Deborah Penet; Doris Popovic; Emmanouil Dermitzakis; Griffin White; Henri Pegeot; Ioannis Xenarios; Ivan Topolsky; Jay Tracy; Katharina Jahn; Keith Harshman; Lara Fuhrmann; Laura Neff; Lennart Opitz; Lorenzo Cerutti; Maria Domenica Moccia; Maurice Redondo; Niko Beerenwinkel; Noemie Santamaria de Souza; Olivier Kobel; Philipp Jablonski; Ralph Schlapbach; Sarah Nadeau; Simon Gruter; Sophie Seidel; Tanja Stadler; Timothy Sykes
EPI_ISL_693831, EPI_ISL_1750667, EPI_ISL_2307734	Viollier AG	Department of Biosystems Science and Engineering, ETH Zürich	Chaoran Chen; Christian Beisel; Christiane Beckmann; Christoph Noppen; David Dreifuss; Deborah Penet; Elodie Burcklen; Emmanouil Dermitzakis; Henri Pegeot; Ina Nissen; Ioannis Xenarios; Ivan Topolsky; Katharina Jahn; Keith Harshman; Lara Fuhrmann; Lorenzo Cerutti; Maurice Redondo; Mirjam Feldkamp; Natascha Santacroce; Niko Beerenwinkel; Noemie Santamaria de Souza; Olivier Kobel; Pedro Ferreira; Philipp Jablonski; Rebecca Denes; Sarah Nadeau; Sophie Seidel; Susana Posada-Céspedes; Tanja Stadler; Tobias Schär
EPI_ISL_1758089, EPI_ISL_1762052, EPI_ISL_1762053, EPI_ISL_1762054, EPI_ISL_1762056, EPI_ISL_1762057, EPI_ISL_1762058, EPI_ISL_1762059, EPI_ISL_1762060, EPI_ISL_1762061, EPI_ISL_1762062, EPI_ISL_1762063, EPI_ISL_1762064, EPI_ISL_1762065, EPI_ISL_1762066, EPI_ISL_1762067, EPI_ISL_1762068, EPI_ISL_1762069, EPI_ISL_1762070, EPI_ISL_1762071, EPI_ISL_1762072, EPI_ISL_1762073, EPI_ISL_1762074, EPI_ISL_1762075, EPI_ISL_1762076, EPI_ISL_1762077, EPI_ISL_1762078, EPI_ISL_1762080, EPI_ISL_1762081, EPI_ISL_1762082, EPI_ISL_1762083, EPI_ISL_1762084, EPI_ISL_1762086, EPI_ISL_1762087, EPI_ISL_1762088, EPI_ISL_1764144, EPI_ISL_1764145, EPI_ISL_1764146, EPI_ISL_1788083, EPI_ISL_1972305, EPI_ISL_1972306, EPI_ISL_1972320, EPI_ISL_1972325, EPI_ISL_2135847			
see above	Viral Respiratory Lab, National Institute for Biomedical Research (INRB)	Pathogen Sequencing Lab, National Institute for Biomedical Research (INRB)	Allison Black; Amuri Aziza; Andrew Rambaut; Catherine Pratt; Eddy Kinganda-Lusamaki; Edith Nkwembe; Emmanuel Lokilo Lofiko; Francisca Muyembe Mawete; Ian Goodfellow; James Hadfield; Jean Claude Makangara; Jean-Jacques Muyembe Tamfum; Josh Quick; Kristian Andersen; Matthias Pauthner; Michael Wiley; Nick Loman; Placide Mbala-Kingebeni; Steve Ahuka-Mundeke; Trevor Bedford
EPI_ISL_918371	Virology Unit, Institut Pasteur du Cambodge	Virology Unit, Institut Pasteur du Cambodge	Chau Darapheak; Chin Savuth; Erik A Karlsson; Etienne Simon-Loriere; Kraing Sidonn; Ly Sovann; Sokhoun Yann; Veasna Duong; Yi Sengdoeurn
EPI_ISL_1400539	WHO National Influenza Centre Russian Federation	WHO National Influenza Centre Russian Federation	Andrey Komissarov; Anna Ivanova; Artem Fadeev; Daria Danilenko; Dmitry Bazhenov; Dmitry Lioznov; Elena Nabieva; Georgii Bazykin; Ksenia Safina; Kseniya Komissarova; Mikhail Bakaev
EPI_ISL_2365407, EPI_ISL_2365408, EPI_ISL_2365409, EPI_ISL_2365410, EPI_ISL_2365411, EPI_ISL_2365412, EPI_ISL_2365413			
see above	WWF Bayanga field laboratory	Robert Koch Institute	F. H. Leendertz; F. S. Niatou-Singa; M. Ulrich; S. Calvignac-Spencer; T. B. Tombolomako; T. Fuh-Neba; U. Vickos
EPI_ISL_2365414, EPI_ISL_2365415, EPI_ISL_2365416, EPI_ISL_2365417	WWF Bayanga field laboratory	WWF Bayanga field laboratory	F. H. Leendertz; F. S. Niatou-Singa; M. Ulrich; S. Calvignac-Spencer; T. B. Tombolomako; T. Fuh-Neba; U. Vickos
EPI_ISL_416538	Wellington Hospital	Institute of Environmental Science and Research (ESR)	New Zealand; Newtown; Riddiford Street; Wellington 6021; Wellington Hospital; Wellington SCL
EPI_ISL_1255144, EPI_ISL_1255271	West African Centre for Cell Biology of Infectious Pathogens (WACCBIP), University of Ghana, Accra, Ghana	West African Centre for Cell Biology of Infectious Pathogens (WACCBIP), University of Ghana, Volta Road, Legon-Accra, Ghana	; Abdoulaye B Diallo; Abdul-Karim Abass; Aisha Mohammed; Benjamin Demah Nuertey; Collins M. Morang'a; Dam Kenneth Mibut; Dominic S.Y. Amuzu; Emmanuella Amoako4; Evelyn B. Quansah; Frederick Kumi-Ansah; Frederick Tei-Maya; Gordon A Awandare; Joyce M. Ngoi; Kesego Tapela; Lucas N. Amenga-Etego; Nelson Kibinge; Oliver D Boakye5; Peter K Quashie; Philip M. Soglo; Samirah Saiid; Samuel Kaba Akoriyea; Theophilus Odoom; Vanessa Magnussen; Vincent Appiah; Yaw Bediako
EPI_ISL_1967489	Wyoming Public Health Laboratory	Wyoming Public Health Laboratory	Ashley Norberg; Brian Dominguez; Brittany Oher; Cari Sloma; Channing Weber; Chayse Rowley; Elliot Thomasson; Jim Mildenberger; Marley Goetz; Sam Britz; Taylor Fearing; and Rob Christensen
EPI_ISL_1565237	amedes MVZ Hannover	Robert Koch Institute	
EPI_ISL_2421124, EPI_ISL_2562034	cerballiance-IDF	Cerba lab	Aude Lessenne; Bénédicte Roquebert; Emmanuel Lecorche; Kader Merah; Laura Verdurme; Patrice Herisson; Sabine Trombert-Paolantoni; Stéphanie Haïm-Boukobza; Thierry Collin
EPI_ISL_2600376	cerballiance-centre val de loire	Cerba lab	Aude Lessenne; Bénédicte Roquebert; Emmanuel Lecorche; Kader Merah; Laura Verdurme; Patrice Herisson; Sabine Trombert-Paolantoni; Stéphanie Haïm-Boukobza; Thierry Collin
EPI_ISL_2248995, EPI_ISL_2248996, EPI_ISL_2362120, EPI_ISL_2362121, EPI_ISL_2362122, EPI_ISL_2362123, EPI_ISL_2362124, EPI_ISL_2362125, EPI_ISL_2362126, EPI_ISL_2362127, EPI_ISL_2362128, EPI_ISL_2362129, EPI_ISL_2362130, EPI_ISL_2362131, EPI_ISL_2362132			
see above	unknown	Instituto Nacional de Saude (INSA)	Borges et al
